# Applications of electromyography in Amyotrophic Lateral Sclerosis: A systematic review

**DOI:** 10.1371/journal.pone.0350029

**Published:** 2026-06-22

**Authors:** Ana Paula Mendonça Fernandes, Luiz Henrique Bertucci Borges, Ledycnarf Januário de Holanda, Bruno Henrique e Silva Bezerra, Anna Clara Sales Miranda Lopes, Maria Clara Fernandes da Silva, Ricardo A. de M. Valentim, Laurent Bougrain, Gabriel Alves Mendes Vasiljevic, Ana Raquel Rodrigues Lindquist

**Affiliations:** 1 Department of Physical Therapy, Federal University of Rio Grande do Norte, Natal, Brazil; 2 Laboratory of Technological Innovation in Health, Federal University of Rio Grande do Norte, Natal, Brazil; 3 Edmond and Lily Safra International Institute of Neuroscience, Santos Dumont Institute (ISD), Macaíba, Brazil; 4 Bloorview Research Institute, Holland Bloorview Kids Rehabilitation Hospital, Toronto, Canada; 5 Institute of Biomedical Engineering (BME), University of Toronto, Toronto, Canada; 6 Department of Biomedical Engineering, Federal University of Rio Grande do Norte, Natal, Brazil; 7 Department of Complex System, Intelligence and Robotics at Loria, University of Lorraine, Nancy, France; Roma Tre University: Universita degli Studi Roma Tre, ITALY

## Abstract

This systematic review examined the use of surface electromyography (sEMG) for the neuromuscular assessment of individuals with Amyotrophic Lateral Sclerosis (ALS), focusing on clinical parameters, the muscle groups evaluated, acquisition protocols, technical properties of the recording systems, integration with other technologies, and signal processing strategies. We included observational studies that applied sEMG to individuals diagnosed with ALS, with or without comparison to healthy controls, and without restrictions on publication year. The analyses included signals recorded at rest and during voluntary contractions, with or without the use of biofeedback. Most studies employed conventional or high-density surface electrodes, with sampling frequencies ranging from 500 Hz to 3000 Hz. The results showed that the primary parameters assessed were muscle fatigue, fasciculation patterns, the number of motor units (MUNE/MUNIX), motor unit firing rates, and signal complexity. These parameters demonstrated sensitivity to disease progression and may contribute to early diagnosis, phenotypic stratification, and functional monitoring of ALS. Additionally, the studies highlighted the increasing use of advanced computational approaches, such as machine learning, for feature extraction and automated classification. In conclusion, sEMG is a promising tool for functional assessment in ALS, with the potential to improve diagnostic accuracy and support new therapeutic strategies based on electrophysiological biomarkers. However, despite technological advances, the included studies displayed substantial methodological heterogeneity and limited protocol standardization. Integration with other neurophysiological modalities also remains underexplored, despite its significant clinical potential.

## Introduction

Amyotrophic Lateral Sclerosis (ALS) is a progressive neurodegenerative disease with a complex pathophysiology that affects the central and peripheral nervous systems [1]. This disease is characterized by the gradual degeneration of upper and lower motor neurons, leading to weakness, muscle atrophy, paralysis, and consequent loss of function [2]. As the disease progresses, the diaphragm and accessory breathing muscles are compromised, significantly reducing patients’ life expectancy to approximately 2 to 5 years after diagnosis, with respiratory failure being the main cause of death [1,3].

There is currently no cure for ALS, and the treatments available aim to preserve, as far as possible, the functionality and quality of life of the affected person throughout the progression of the disease [4]. Multi-professional support has proven to be the most effective approach to achieving this goal, integrating different specialties and assistive technologies [4]. Among these technologies is electromyography (EMG), which can be applied invasively or through Surface Electromyography (sEMG). This technique records and analyzes the electrical signals generated by the activation of motor neurons in their corresponding muscle fibers [5], making it a promising tool for monitoring the muscular health of individuals with ALS [6].

sEMG allows the assessment of important parameters such as muscle fatigue [7], the recruitment of motor units [8], and the presence of fasciculations [9]. In addition, it can be used as a biosignal to control assistive technologies, helping to maintain the functionality of people with ALS [10]. Despite requiring trained professionals and being expensive, this technique offers advantages over other neuromuscular assessment methods, such as dynamometry [11] and invasive EMG [12], since it provides direct data on nerve conduction without the need for intramuscular insertion, making it a safe and effective approach [12].

Currently, there is growing interest in the use of sEMG, due to the analysis of the characteristics of the signals [12]. Previous studies have shown that sEMG is effective in detecting altered muscle activation patterns in individuals with ALS [13,14]. However, despite the evidence supporting their applicability, the protocols for collecting and analyzing electromyographic signals still show great methodological variability [15]. This heterogeneity, combined with the clinical variability of the disease, makes it difficult to define standardized guidelines for obtaining and interpreting sEMG data in this population.

Thus, given the lack of a comprehensive overview of the clinical applicability of sEMG in individuals with ALS, it is necessary to carry out a review that can guide health professionals and researchers as to the most commonly used and recommended procedures for acquiring and processing these signals. Among the issues that need to be clarified are the clinical parameters assessed, the most frequently analyzed muscle groups, the activities performed during collection, rest times, the instruments used to assess the sample, the use of biofeedback, the physical properties of the electrodes and equipment, as well as aspects related to the calibration, acquisition, recording, amplification, filtering, normalization, and processing of electromyographic signals.

The standardization of sEMG acquisition and reporting procedures is essential to ensure reproducibility and comparability across studies. In this context, international guidelines have been developed to promote methodological best practices. The European project *Surface EMG for Non-Invasive Assessment of Muscles* (SENIAM) [16] was a pioneer in proposing detailed recommendations regarding sensor type, electrode placement, and skin preparation, establishing a common basis for the use of sEMG in clinical and research settings. More recently, the *Consensus for Experimental Design in Electromyography* (CEDE) expanded these recommendations through a series of publications addressing terminology and conceptual standardization [17], the use of high-density surface EMG [18], single motor unit analysis [19], and a comprehensive checklist for the planning and reporting of EMG studies (*CEDE-Check*) [20]. The adoption of these guidelines is particularly relevant for studies on ALS, as it enhances methodological rigor, transparency, and comparability between results, thereby contributing to the identification of neuromuscular biomarkers and strengthening the clinical applicability of sEMG in this population.

As a result, professionals involved in the diagnosis, monitoring, and rehabilitation of people with ALS will be able to rely on knowledge to guide the use of sEMG in the care of this population.

## Materials and methods

The systematic review was conducted and structured following the guidelines of the Preferred Reporting Items for Systematic Reviews and Meta-Analyses (PRISMA) [21]. The evaluation began in July 2024 and was completed in February 2025. Accordingly, the analysis encompassed studies and elements concerning the use of sEMG—whether or not combined with validated scales—in individuals with ALS or about specific disease outcomes, including data on processing methods, intervention and analysis procedures, participant profiles, and the clinical variables assessed.

The review protocol entitled *Electromyography as a tool to motion analysis for people with Amyotrophic Lateral Sclerosis: A protocol for a systematic review* was registered in the International Prospective Register of Systematic Reviews (PROSPERO) under ID CRD42023465596 and published in the journal *Plos ONE* [22], on May 28, 2024, and can be accessed at https://doi.org/10.1371/journal.pone.0302479.

### Research questions

Following the methodological rigor described in the [22] protocol, all the relevant studies were selected to answer the study’s research questions, which investigate different aspects of the use of sEMG in the assessment of individuals with ALS, considering clinical parameters, methodologies used and integration with other technologies, as shown in the Table 1.

**Table 1:** Research questions.

Aspects	Research Questions
Motor parameters	RQ1: Which motor parameters, such as fatigue, fasciculations, paresis, or plegia, have been assessed using sEMG in individuals diagnosed with ALS?
Participant characteristics	RQ2: What participant characteristics were reported?
Targeted muscles	RQ3: Which muscle groups were evaluated using sEMG? Did the evaluation include muscles of the upper limbs, lower limbs, trunk, cervical region, or head and neck?
Evaluation protocol	RQ4: How was the sEMG-based evaluation performed? Which activities were employed, what was the duration of data collection, and what rest periods were adopted?
Equipment and acquisition parameters	RQ5: What equipment and parameters were used for sEMG acquisition (e.g., electrode type, sampling frequency, portability, and data transmission method)?
sEMG signal processing	RQ6: What processing techniques were applied to the sEMG signal? Which software and analysis algorithms were used?
Integration with other technologies	RQ7: Was sEMG used in conjunction with other data collection technologies, such as electroencephalography (EEG), inertial sensors, or neuroimaging?
Use of biofeedback	RQ8: Was sEMG used as a biofeedback tool at any stage of the study? If so, at what point in the evaluation or follow-up was it applied?
Intervention and rehabilitation strategies	RQ9: Which intervention or rehabilitation strategies were associated with sEMG analysis? Have any studies used sEMG as a support tool for motor rehabilitation?
Outcomes and processing methods	RQ10: What outcomes were observed in the analyzed motor parameters? Which sEMG processing methods demonstrated the best performance and applicability?

### Eligibility criteria

The eligibility criteria were previously defined and recorded in the protocol for this systematic review [22]. The selection of studies was guided by the PICOTS strategy (Participants, Interventions, Comparisons, Outcomes, Period, and Type of Study) [23], contemplating:

- **Participants:** individuals with a confirmed diagnosis of ALS, according to the revised El Escorial criteria [24].
- **Interventions:** use of sEMG to characterize motor symptoms, alone or combined with clinical scales or assistive technologies.
- **Comparisons:** different signal analysis approaches (time, frequency, or both), as well as comparing sEMG device configurations.
- **Outcomes:** motor parameters obtained through sEMG. Studies focusing exclusively on respiratory muscles, cognitive, or bulbar function were excluded.
- **Evaluation periods:** baseline, post-intervention (short term) and follow-up (medium or long term).
- **Eligible study types:** observational (cross-sectional and longitudinal), quasi-experimental and experimental studies, including randomized and non-randomized clinical trials.

### Electronic research

The articles were selected through a systematic search conducted in various electronic databases, including MEDLINE (via PubMed), Web of Science, Embase, IEEE Xplore, Scopus, Google Scholar, SciELO, PEDro, Cochrane CENTRAL, and LILACS. Ongoing or unpublished clinical trials were identified through the ClinicalTrials.gov and World Health Organization International Clinical Trials Registry Platform. To complement the search, the reference lists of the included studies were manually analyzed, as well as the proceedings of relevant conferences, to locate potentially unindexed grey literature.

### Search strategy

The search strategy used in this review was previously recorded and is detailed in the research protocol [22]. To ensure that the present manuscript is self-contained, we summarize the full search approach below. The strategy was developed using a combination of controlled descriptors (MeSH and DeCS) and free terms related to surface electromyography, Amyotrophic Lateral Sclerosis, clinical motor signs and symptoms, physical rehabilitation, and body segments.

Keywords and synonyms were grouped into broad conceptual categories—body segment, physical rehabilitation, clinical data, electromyography/mechanomyography, and ALS terminology—as presented in Table 2. Our multidisciplinary team identified relevant descriptors and synonyms, which were then adapted to the syntax and indexing structure of each database. Boolean operators (“AND”, “OR”), truncation symbols, and field tags were applied as appropriate.

**Table 2:** Main concepts and related keywords.

Concept	Matching Keywords
Body segment	Upper extremity, upper limb, trunk musculature, cervical, lower extremity, lower limb, hand, elbow, wrist, shoulder, forearm, arm, fingers, foot, ankle, knee, hip.
Physical rehabilitation	Physical rehabilitation, motor rehabilitation, physical medicine, telerehabilitation, physiotherapy, functional outcome.
Clinical data	Motor signs and symptoms, fasciculations, cramps, weakness, fatigue, stiffness, spasticity, clonus, hypertonia, hyperreflexia.
Electromyography	sEMG, EMG, muscle activity, muscle activation.
Amyotrophic lateral sclerosis	ALS, amyotrophic lateral sclerosis, Charcot disease, Lou Gehrig’s disease, motor neuron disease, ALS-related parkinsonism-dementia complex, Guam disease, amyotrophic lateral sclerosis with dementia.

We adopted a sensitive and non-specific search strategy to maximize retrieval of potentially eligible studies. Therefore, search terms were intentionally broad, and irrelevant articles were excluded during the selection phase. No restrictions were applied regarding language or year of publication.

### Study selection

Three independent reviewers (APMF, MCFS, ACSML) analyzed the titles and abstracts to determine the eligibility of the studies. Any disagreements were resolved with the participation of a fourth reviewer (BHSB). The selected studies were imported into the Rayyan software [25], making it easier to exclude duplicates and apply the inclusion and exclusion criteria. In cases of doubt, the full text was obtained for a more detailed evaluation. Studies that did not meet the criteria of the review protocol concerning the type of study, participants, intervention, comparison group and study design were excluded.

To ensure that only relevant articles were included in the review, the following Selection Criteria (SC) were established when reading the title and abstract:

- SC1: Articles published in peer-reviewed scientific journals or peer-reviewed scientific conference proceedings;
- SC2: Studies using sEMG as an analytical tool in the investigation of ALS;
- SC3: Studies that have applied sEMG signal processing and analysis methods to the functional assessment of ALS patients;

The selection criteria were applied as a first filter to gather only relevant studies. CS1 ensures the reliability of the articles selected, guaranteeing scientific rigor in the description of methods, results, and analysis. CS2 and CS3 reinforce the relationship between the studies and the scope of the review, focusing on neuromuscular analysis in ALS patients.

When the abstract did not provide enough information about the focus of the research, the article was temporarily kept for full reading. At this initial stage, the methodological quality of the studies was not assessed. The selected articles were then applied to the Exclusion Criteria (EC):

- EC1: The study did not investigate the use of sEMG signals to collect neuromuscular data from people with ALS or to identify clinical signs and symptoms;
- EC2: The study exclusively used a biological signal other than the neuromuscular signal;
- EC3: The study did not collect data from people diagnosed with ALS.

If an article met any of the Exclusion Criteria, it was eliminated from the review. The remaining articles were then assessed for Inclusion Criteria (IC):

- IC1: The study used sEMG to collect data from people with ALS;
- IC2: The study featured neuromuscular processing and analysis to assess clinical signs in people with ALS.

If an article did not meet any of the Inclusion Criteria, it was excluded. After this screening, the selected articles were subjected to a detailed assessment of methodological quality and risk of bias to determine their final inclusion in the systematic review.

### Data extraction

Data extraction was conducted by five independent evaluators (APMF, MCFS, ACSML, LHBB, BHSB), based on a standardized form previously defined in the study protocol [22]. Any disagreements were initially resolved by discussion between reviewers or, if necessary, with the intervention of a sixth reviewer (GAMV).

The information extracted included general data on the studies (year, authors, location, evaluator group), characteristics of the participants (number, age, time since onset of symptoms and diagnosis), details of the control groups, and information on the instruments and protocols used. Technical aspects of sEMG signal collection, such as electrodes, sampling frequency, muscles analyzed, type of motor tasks, and integration with other technologies, were also recorded.

The sEMG signal processing was described in detail, including the filters applied, analysis domains (time, frequency, and time-frequency), computational tools, and algorithms used. The main findings were organized into three axes: clinical data, sEMG signal processing, and the effects of the interventions.

In cases of incomplete data, the corresponding authors of the included studies were contacted to obtain the missing information, as provided for in the protocol.

### Data analysis

#### Methodological quality.

The methodological quality of the studies was evaluated using the GRADE (Grading of Recommendations Assessment, Development, and Evaluation) system [26]. In this review, the GRADE assessment was used not only to classify the certainty of the evidence but also as an eligibility criterion.

Studies presenting very low-quality evidence—characterized by serious methodological limitations, inconsistency of results, indirectness, imprecision, or strong suspicion of publication bias—were excluded from the final synthesis. Only studies rated as moderate or high quality were included.

The GRADE assessment considered factors such as study design, methodological limitations, inconsistency of findings, indirect evidence, imprecision of estimates, publication bias, magnitude of effect, dose–response gradient, and residual confounding [27]. All evaluations were conducted by two independent assessors (MCFS and ACSML), and any disagreements were resolved by a third reviewer (APMF).

#### Risk of bias.

Risk of bias was assessed using the Prediction model Risk Of Bias Assessment Tool (PROBAST) [28]. As with the GRADE assessment, PROBAST ratings were also used as part of the study selection criteria.

Studies classified as having a high risk of bias in any of the four PROBAST domains were excluded. Only studies rated as low risk of bias were included in the quantitative synthesis.

The assessment was performed independently by two reviewers (BHSB and ACSML), with discrepancies resolved through discussion with a third reviewer (APMF) to ensure transparency and consistency in decision-making.

## Results

The results of this review are presented according to the research questions, covering the clinical parameters assessed, the muscle groups investigated, the signal collection and analysis protocols, the comparison with control groups, the use of biofeedback, integration with complementary technologies, data processing methods, and the main clinical outcomes and processing methods identified.

### Study selection

A total of 30,478 records were identified. After removing duplicates, 23,245 articles were screened by title and abstract, with 531 selected for full-text review. Following eligibility assessment and methodological quality evaluation, 34 studies were included in the quantitative synthesis.

**Fig 1:**
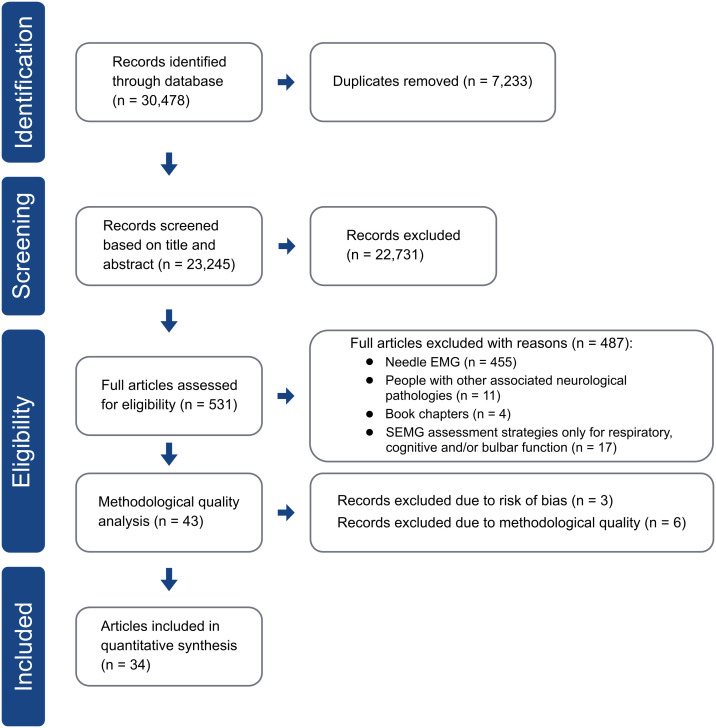
Flowchart of the study selection process according to PRISMA guidelines.

The summary of this process is illustrated in the PRISMA flow diagram in Fig 1.

### Date and place of publication

**Fig 2:**
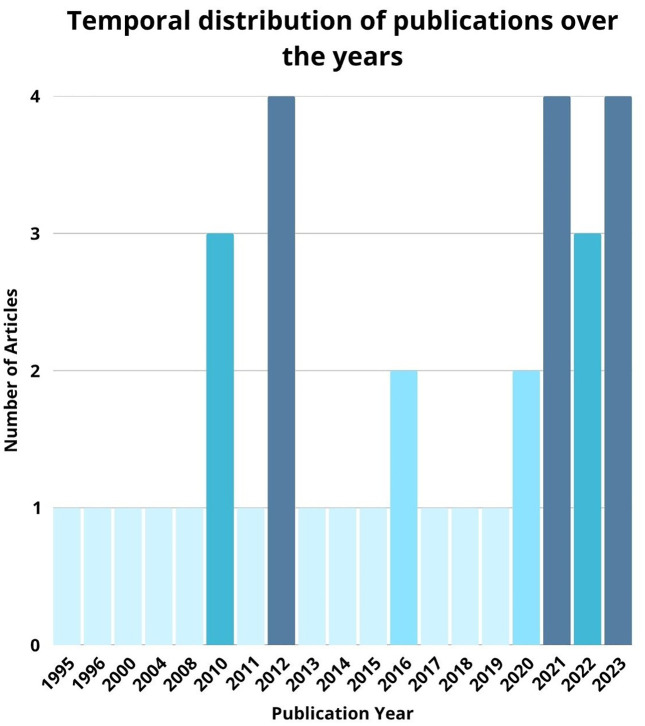
Distribution of included studies by year of publication.

Fig 2 below shows the results of the temporal analysis of the publications included in the review, illustrating the distribution of studies over the years.

The studies included in this review were published between 1995 and 2023. The earliest works, from the mid-1990s, originated in the United States, such as the studies by Felice et al., [29] and Bromberg et al., [30]. Throughout the 2000s, research output remained moderate, with contributions from Kent et al., [31] in the United States, Sanjak et al., [32] in China, and Kleine et al., [33] in the Netherlands. However, from 2010 onwards, there was a noticeable increase in scientific research, with significant contributions from the Netherlands [6,34,35], Australia [36], Switzerland [37,38], South Korea [39,40], China [9,13,41,42], Brazil [43], and the United States [44,45]. In the past five years, this growth became even more evident, especially with new publications from the United Kingdom [46–51], Portugal [52–54], France [55], Spain [56], Japan [14,57], and again the United States [58]. This trend highlights the growing global interest in sEMG applications in the context of ALS.

### Clinical data identified

*RQ1: Which motor parameters, such as fatigue, fasciculations, paresis, or plegia, have been assessed using sEMG in individuals diagnosed with ALS?*

The studies included in this review addressed clinical parameters such as fatigue, fasciculations, muscle weakness, loss of motor units, muscle coactivation, recruitment patterns, and changes in neuronal excitability, presented in the S1 Table – Motor Clinical Parameters Evaluated in the Included Studies.

The **fatigue**, both peripheral and central, has been widely investigated and assessed through changes in strength, activation patterns, and functional performance [31,32,37,39,43,56].

The **muscle fasciculations** were characterized in terms of frequency, morphology, amplitude, firing pattern, neuronal origin, and electromechanical latency, using different detection approaches and tools [9,33,34,44–49,51].

**Muscle weakness** was analyzed using measures such as maximum voluntary strength, the Medical Research Council (MRC) Muscle Strength Scale, and functional decline [6,30,35,38,43,58].

The **loss of motor units** was investigated using techniques such as MUNE and MUNIX, with a focus on analyzing the morphology of motor unit action potentials (MUAPs) and neuronal hyperexcitability [13,29,35–37,57].

Aspects such as **muscle coactivation** and **activation rhythm** were also explored [56], as well as **motor unit firing patterns** [14,42,50], the **variability of the interspike interval**, and the **dispersion of the innervation zone** [44], as well as **reduction in recruitment** and **overlapping of action potentials** [13,42].

In addition, synchrony-related measures derived from sEMG were identified, particularly those based on coherence analysis. Intermuscular coherence (IMC) was used to quantify the degree of common oscillatory drive and synchronization between muscle pairs, especially in the beta-band frequency range, providing indirect information about corticospinal and upper motor neuron integrity. Studies reported reduced coherence values in individuals with ALS compared to healthy controls, reflecting impaired neural coupling and altered motor control strategies [52].

Changes in **the morphology of sEMG signals** aiming at automated diagnosis were also analyzed [53], as well as the application of the **cutaneous silent period (CutSP)** as an indirect measure of upper motor neuron dysfunction [54]. Finally, **characteristics of upper and lower motor neurons** were explored based on muscle activation patterns [52,55].

### Characteristics of the participants

*RQ2: What participant characteristics were reported?*

The data describing the characteristics of the participants is presented in the S2 Table – Participant Characteristics. The average age of ALS patients varied between the studies, ranging from approximately 58–65 years. Some reported averages with standard deviation, such as 59,3 ± 11,3 years [38], 58 ± 12 years [36], 60,1 ± 11,2 years [40], 62,3 ± 8,5 years [43], 64,9 ± 9,6 years [58] and 65,4 ± 10,3 years [14]; while others showed simple average values or ranges, such as 60,5 years (55,2–65,4) [49] and 59,5 years [54]. Additional studies have indicated averages of around 64 years [6,33,35].

The average duration since the onset of symptoms was also variable, with reports ranging from around 9–28 months. Some studies have indicated values such as 13,4 ± 5,8 months [38], 8,9 ± 7,8 months [40], 17,9 ± 12,3 months [58], 19 ± 11 months [43], 23 months [49], about 20,25 months [36], and median 28 months (IQR: 17–49) [51]. The average time to diagnosis was 11,4 ± 4,4 months [14] and 13,2 months (IQR: 7,6–19,8) [49]. Many studies, however, did not provide this information.

Various instruments were used for clinical assessment. The ALS Functional Scale-Revised (ALSFRS-R) has been widely used [6,14,35,37–40,47,49,51,54,57,58]. The MRC muscle strength scale was also widely used [6,35,39,43,46,51,54].

Other methods included the Forced Vital Capacity (FVC) [49] and the Ashworth scale for spasticity [54]. Some studies have proposed specific classifications of participants, such as the distinction between predominantly upper or lower motor neuron involvement [55], and categorization according to the type of onset of ALS [34].

### Targeted muscles

*RQ3: Which muscle groups were evaluated using sEMG? Did the evaluation include muscles of the upper limbs, lower limbs, trunk, cervical region, or head and neck?*

**Fig 3:**
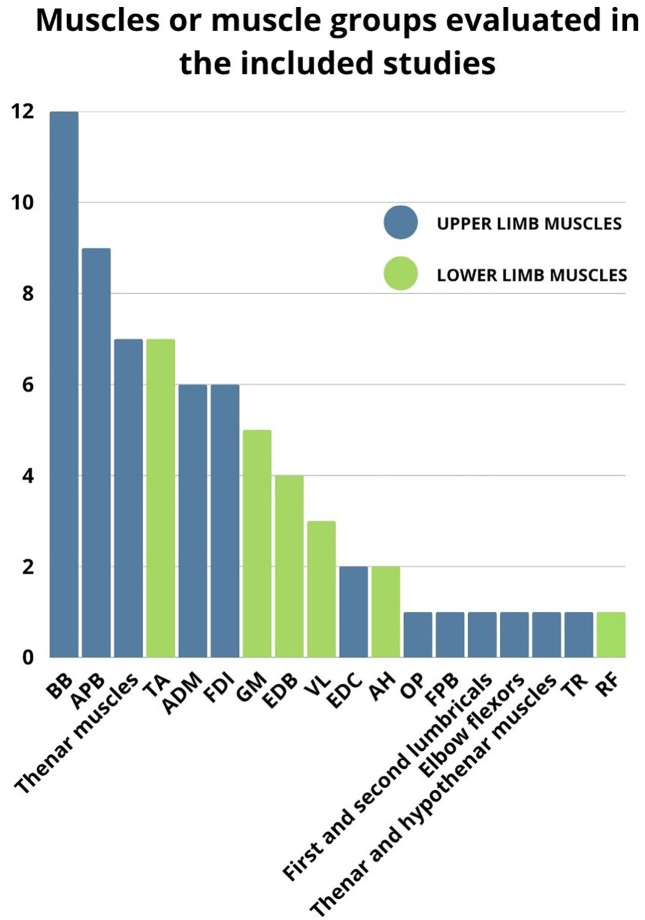
Distribution of the muscles analyzed. **Abbreviations:** ADM: Abductor Digiti Minimi; AH: Abductor Hallucis; APB: Abductor Pollicis Brevis; BB: Biceps Brachii; EDB: Extensor Digitorum Brevis; EDC: Extensor Digitorum Communis; FDI: First Dorsal Interosseous; FPB: Flexor Pollicis Brevis; GM: Medial Gastrocnemius; OP: Opponens Pollicis; RF: Rectus Femoris; TA: Tibialis Anterior; TR: Triceps Brachii; VL: Vastus Lateralis.

Information on all muscles assessed is provided in S3 Table – Muscles or muscle groups evaluated in the included studies and Fig 3.

In the upper limbs, the most commonly evaluated muscle was the abductor pollicis brevis (APB), frequently analyzed across studies using different assessment techniques and clinical parameters [6,9,29,36,37,39–41,43,47,54]. The abductor digiti minimi was also widely studied, mainly in investigations focused on motor units and signal variability [36,37,39,40,43]. The first dorsal interosseous was included in analyses of signal morphology, pattern classification, and machine learning approaches [9,13,40,42,52,53]. Studies evaluating the extensor digitorum focused on muscle recruitment and activation [36,37,47,52,53]. The biceps brachii was investigated in terms of neuronal excitability, functional loss, and fasciculations [9,33,37,44,47,50,54,56], while the triceps brachii appeared less frequently, mainly in studies of coactivation and fatigue [56]. Muscles from the thenar and hypothenar groups were commonly used for MUNE/MUNIX and excitability characterization [9,29,30,35,41], and the lumbricals were assessed in longitudinal investigations [6].

In the lower limbs, the tibialis anterior (TA) was the most frequently examined muscle, especially in studies assessing strength, fatigue, and recruitment [31,32,37,43,54–56]. The medial gastrocnemius was analyzed primarily in studies focusing on fasciculations and action potential morphology [34,46,47,51]. The vastus lateralis appeared in analyses of firing patterns and motor units [14,33,57]. The abductor hallucis (AH) was included in studies estimating motor unit loss [37], while the extensor digitorum brevis (EDB) was examined in investigations of measurement reproducibility [36,37,47]. The rectus femoris (RF) was used in studies exploring coactivation and fatigue [56].

Although limb muscles predominated, a small number of studies evaluated trunk and cervical muscles [41,52]. These regions present additional challenges for sEMG analysis due to multilayered and deeper musculature, greater influence of respiratory and postural adjustments on the signal, and, in some cases, susceptibility to ECG artifacts, particularly when electrodes are placed near the thoracic cavity. These factors can reduce signal-to-noise ratio and increase variability compared to limb recordings. No studies were identified that directly assessed muscles of the head or neck.

### Evaluation

*RQ4: How was the evaluation performed using sEMG? Which activities were employed, what was the duration of data collection, and what rest periods were adopted?*

During the collection of sEMG signals in the studies analyzed, we observed a wide variety of procedures involving different muscle activation and rest strategies, depending on the methodological objectives. This information is present in S4 Table – Data collection activities and rest/collection time in the included studies.

Several studies have collected data with participants at absolute rest, without any kind of voluntary contraction. This approach aimed to record fasciculations and spontaneous activity, and was used by Kleine et al., 2008 [33], Bashford et al., 2019 [46], Zhang et al., 2014 [45], Zhou et al., (2012, 2011) [9,41] Wannop et al., 2021 [49], Bashford et al., 2020a [47], Bashford et al., 2020b [48], Planinc et al., 2023 and [51], with recording sessions varying between 3 and 30 minutes per muscle. In these protocols, rest intervals were generally not specified because recordings were continuous.

Other studies have chosen to collect data during voluntary isometric contractions at different intensities. In general, the participants were instructed to perform contractions at minimum, intermediate and maximum levels of force, with visual or biofeedback control, according to Boekestein’s studies et al., 2012 [35], Ahn et al., 2010 [39], Kim et al., 2016 [40], Escorcio et al., 2016 [43], Neuwirth et al., 2010 [37], Neuwirth et al., 2017 [38], Alarcon et al., 2022 [56], Weddell et al., 2021 [50], Quintão et al., 2021 [52], Sanjak et al., 2004 [32], Chen et al., 2018 [42], Zhang et al., 2013 [13], Nishikawa et al., 2022 [14] and Noto et al., 2023 [57]. The protocols included progressive activation ramps, sustained contractions for up to 60 seconds, and rest periods typically ranging from 10 to 60 seconds when reported; however, several studies did not specify the exact rest duration, describing them only as “adequate” to prevent fatigue.

Some studies applied specific functional tasks during collection. For example, Antunes et al., 2023 [53] asked for coordinated movements of isometric lifting of the index finger against the other fingers, alternating with relaxation periods, although the exact duration of these rest periods was not reported. Quintão et al., 2021 [52] used cycles of contraction and rest of the extensor digitorum muscle, with approximately 10 seconds of rest between cycles. Saidane et al., 2021 [55] recorded electromyographic activity while the participants walked barefoot for five meters, a protocol that did not involve structured rest intervals.

Median or ulnar nerve stimulation techniques were applied to obtain compound potentials (CMAP) and estimates of the number of motor units (MUNE or MUNIX), as in Felice et al., 1995 [29], Baumann et al., 2012 [36], Nandedkar et al., 2022 [58], and Boekestein et al., 2012 [35]. The protocols included variations in stimulus intensity, number of repetitions, and electrode location, ensuring reproducible parameters that were sensitive to the progression of the disease. Rest periods between stimulations were generally brief (a few seconds), but most studies did not quantify them explicitly.

Finally, studies focusing on fatigue or prolonged recordings have adopted more extensive protocols. Kent et al., 2000 [31] performed intermittent isometric dorsiflexion exercises for 25 minutes, with contraction and relaxation cycles, although the exact duration of the relaxation phases was not reported. Alarcon et al., 2022 [56] evaluated muscle response during bilateral maximal contractions until fatigue, with individually determined rest intervals that were not numerically defined. Jahanmiri et al., 2015 [44] collected data at rest and during light contraction, with sessions between 10 and 30 minutes. Zhang et al., 2013 [13] and Chen et al., 2018 [42] performed isometric contractions with a minimum duration of 2–5 seconds and rest periods of 10–15 seconds between attempts.

### sEMG properties

*RQ5: What equipment and parameters were used for sEMG acquisition (e.g., electrode type, sampling frequency, portability, and data transmission method)?*

The data describing the technical properties of the sEMG signals are presented in the S5 Table - sEMG device characteristics.

About the type of electrode, most studies have used surface electrodes, including self-adhesive, pre-frozen, and high-density models. Self-adhesive cut Electrocardiogram (ECG) electrodes, pre-frozen 20 mm diameter electrodes, disposable electrodes with diameters between 10 and 15 mm, 10×30 mm models, and 22×24 mm Ambu Blue Sensor Tabs were used. [29,36–38,40,43]. Disk electrodes have also been widely applied [39,58].

Several studies have used high-density electrodes (HD-sEMG), such as 8×15 grids with 4 mm spacing and 10×13 matrices with a 5 mm distance between electrodes [6,33–35]. Grids of 64 circular electrodes (8×8), each with a diameter of 4.5 mm, have also been widely used [46–48,50,51].

Few systems have been described as portable. Highlights include the Biosignalsplux, with 8 channels and a built-in filter, and the BTS FreeEMG, with Bluetooth transmission [53,56]. Most used laboratory equipment, such as Biosemi, TMS International BV, OT Bioelettronica, Natus Medical, Synergy, and Keypoint Classic.

As for the method of data transmission, several systems stored the signals locally for offline analysis [33,34,39,58]. In studies with HD-sEMG, it was common to use a wired connection with Refa64 or Refa128 amplifiers [9,41,42,46,51]. Other studies have reported wireless systems via Bluetooth or with on-board acquisition [53,56].

The sampling frequency varied widely. In general, HD-sEMG systems operated at 2048 Hz [6,34,35,46–48,51,57], while frequencies of 2000 Hz were also common [9,13,33,41,42,44]. Intermediate frequencies, such as 1000 Hz, have been reported [52,53,56], and lower values, such as 500 Hz, have also been used [32]. One study highlighted the application of 3000 Hz [54]. Some studies did not specify this information [29,30,36,37].

### sEMG signal analysis

*RQ6: What processing techniques were applied to the sEMG signal? Which software and analysis algorithms were used?*

In S6 Table – Signal processing features in surface sEMG studies, the methods for processing and analyzing the sEMG signals are presented. Analog and digital filters between 10 and 450 Hz, combined with wavelet transforms, fractal analysis, and temporal and spectral feature extraction, were applied to classify signals from ALS patients and healthy controls, using an AdaBoost classifier [53]. A similar strategy was adopted for the extraction of metrics such as multiscale entropy (MSE), Detrended Fluctuation Analysis (DFA), and Lempel-Ziv complexity, feeding classifiers such as Random Forest, AdaBoost, Linear Discriminant Analysis (LDA), and K-Nearest Neighbors (KNN). [52].

The complexity of firing patterns was analyzed using multiscale entropy (MSE) and power spectral density (PSD), focusing on the linearity, Gaussianity, and sparsity of the signals [45]. In addition, complexity quantification was explored using Approximate Entropy (ApEn) and Fast Fourier Transform (FFT) to discriminate spontaneous firing patterns [41].

Time-frequency approaches have also been applied, such as the discrete wavelet transform, the Short Time Fourier Transform (STFT), and cepstrum analysis, combining these techniques with machine learning to classify healthy people and those with ALS, differentiating between upper and lower motor neuron involvement [55]. Extracted characteristics included mean amplitude, RMS, Willison Amplitude (WAMP), sample entropy, and median frequency.

Other studies have focused on the decomposition of high-density signals. The *Automatic Progressive FastICA Peel-Off* (APFP) algorithm was developed for blind source separation, with filters between 10–500 Hz and metrics such as Matching Rate and False Discovery Rate [42]. A peak detection routine was also used, exploring high-density signals and studying muscle fatigue after isometric contractions [56].

The detection and analysis of fasciculations was addressed with the *SPiQE* system, which uses 20–500 Hz band-pass filters, automatic exclusion of noisy channels, and a linear model for correlating fasciculation amplitude and depth [34,46,51].

### Integration with other technologies

*RQ7: Was sEMG used in conjunction with other data collection technologies, such as EEG, inertial sensors, or neuroimaging?*

Our finding reveal that the integration of sEMG with other data collection technologies is still infrequent in research into ALS. This information can be found in the Table 3.

**Table 3:** Studies integrating sEMG with other technologies.

Authors	Integration with Other Devices
Kent-Braun et al., 2000	Magnetic Resonance Spectroscopy (MRS)
Castro et al., 2023	Transcranial Magnetic Stimulation
Sanjak et al., 2004	Force transducer (SM-250, Interface and Advance Force Measurements)
Nishikawa et al., 2022	Custom dynamometer
Planinc et al., 2023	Muscle ultrasonography (MUS)
Noto et al., 2023	Dynamometer

**Abbreviations:** MRS: Magnetic Resonance Spectroscopy; MUS: Muscle Ultrasonography.

sEMG was used in conjunction with magnetic resonance spectroscopy (MRS) to assess central fatigue during isometric exercise, demonstrating an integrated approach between peripheral muscle activity and cerebral metabolic parameters [31]. They have also been associated with muscle ultrasound (MUS) in the investigation of the electromechanical latency of fasciculations [51]. Another example includes the combination with transcranial magnetic stimulation (TMS) to assess upper motor neuron dysfunction [54].

In addition, mechanical support technologies have also been integrated. A force transducer was used to explore the dissociation between mechanical and electromyographic manifestations of fatigue [32], while customized dynamometers made it possible to measure muscle force accurately during signal acquisition [50,57].

### Use of biofeedbacks

*RQ8: Was sEMG used as a biofeedback tool at any stage of the study? If so, at what point in the evaluation or follow-up was it applied?*

The use of biofeedback strategies has been reported as a resource to guarantee signal quality and control contraction force during data capture. Visual and auditory monitoring were used for this purpose, enabling real-time adjustments to muscle contraction [37,39,43]. Providing direct visual feedback of the sEMG signal during collection has also been reported [34].

**Fig 4:**
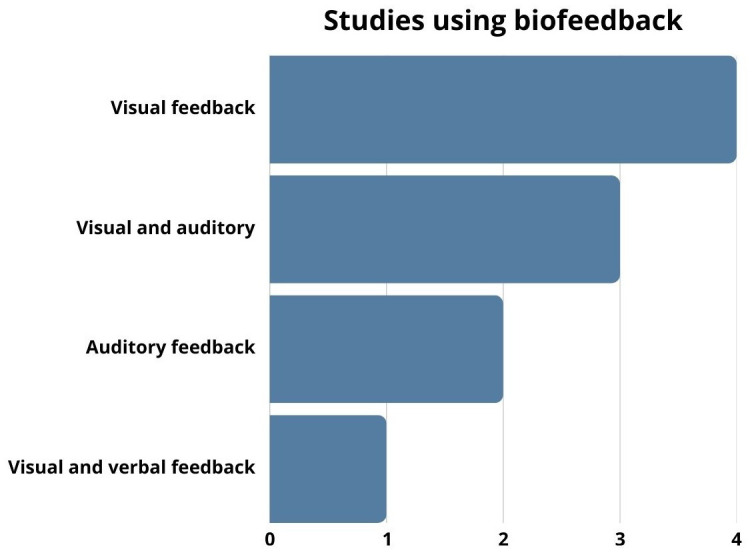
Description of the feedback methods used in the studies.

Other forms of biofeedback include the use of sound signals to guide motor activity [53], combinations of visual and verbal instructions to standardize contractions [50], and real-time visual feedback related to contraction force [57]. Providing visual feedback of the force exerted during isometric tasks showed an association between sEMG and functional performance [31], while the use of auditory feedback was also described during the tests [54]. It is important to emphasize that none of these biofeedback procedures constitute therapeutic or experimental intervention. They were employed exclusively to standardize motor performance and ensure the reliable quality of the sEMG. This data is described in the Fig 4.

### Characteristics of the intervention

*RQ9: Which intervention or rehabilitation strategies were associated with sEMG analysis? Have any studies used sEMG as a support tool for motor rehabilitation?*

The data related to the interventions can be found in the Table 4. Electrical stimulation was used in different protocols involving the collection of sEMG for evaluation purposes. Stimuli on the median nerve and peripheral nerves were applied as part of protocols for estimating motor units, using specific equipment such as the Dantec Model 13L36 and the Nicolet Viking IV [29,36]. Supramaximal stimulation of the peroneal nerve was also performed before and after isometric exercises to investigate central fatigue, with no therapeutic purpose [31]. In addition, cutaneous stimuli were used to induce the cutaneous silent period (CutSP), with a view to functional evaluation [54].

**Table 4:** Summary of studies with interventions.

Authors	Intervention Instrument	Intervention Moment	Description
Felice et al., 1995	Dantec, Model 13L36	During EMG acquisition	Electrical stimulation of the median nerve at multiple forearm sites; S-MUAPs recorded for MUNE estimation.
Baumann et al., 2012	Nicolet Viking IV System for MUNE	During EMG acquisition	Surface electrode stimulation; CMAP recording; Bayesian analysis for motor unit estimation.
Kent-Braun et al., 2000	Supramaximal stimulation of peroneal nerve	During and after exercise	Electrical stimulation (25V above threshold) to assess central and peripheral fatigue.
Castro et al., 2023	Orthodromic cutaneous electrical stimulation	During EMG acquisition	Electrical stimuli applied to the 5th digit and sural nerve to induce Cutaneous Silent Period (CutSP).
Alarcón-Jimenez et al., 2022	Isometric exercise	During EMG acquisition	Five 1-second maximal isometric contractions of the biceps brachii with 5-second rests.

**Abbreviations:** CMAP: Compound Muscle Action Potential; CutSP: Cutaneous Silent Period; EMG: Electromyography; MUNE: Motor Unit Number Estimation; S-MUAPs: Single Motor Unit Action Potentials.

None of the studies analyzed reported the application of sEMG as an active tool in rehabilitation protocols or as a control interface for assistive technologies. The interventions described focused predominantly on neurophysiological analysis.

### Clinical results and processing

*RQ10: What outcomes were observed in the analyzed motor parameters? Which sEMG processing methods demonstrated the best performance and applicability?*

The main clinical motor parameters assessed using sEMG in individuals with ALS were muscle fatigue, the frequency and pattern of fasciculations, the estimated number of motor units (MUNE/MUNIX), and the firing rate of motor units, data presented in the S7 Table – Summary of clinical data and sEMG signal processing outcomes in ALS studies. These parameters have proved effective in differentiating clinical subtypes, characterizing disease progression, and predicting survival, offering objective support for functional assessment and longitudinal monitoring.

Muscle fatigue is predominantly central in ALS, with progressive failure of central activation observed during sustained isometric contractions, correlated with a smaller reduction in phosphocreatine levels [31]. The dissociation between the reduction in muscle strength and the discrete changes in myoelectric signals, observed through the median frequency, also reinforces the central origin of fatigue [32]. Recent studies, such as that by Alarcón et al., 2022 [56], have identified abnormal muscle activation patterns during maximum isometric contraction, highlighting the usefulness of dynamic sEMG in functional assessment.

The frequency and pattern of fasciculations are important biomarkers in ALS. Klein et al., 2008 [33] identified two distinct electromyographic patterns of fasciculations, while the SPiQE system, developed by Bashford et al., 2019 [46], allows automated detection with high accuracy. Subsequent studies by Bashford et al., 2020 [48] have shown that the frequency of fasciculations varies over time, with an initial increase and decline as the disease progresses. Planinc et al., 2023 [51] investigated the depth and latency of fasciculations, and Wannop et al., 2021 [49] introduced the Rate of Change of Fasciculation Frequency (RoCoFF) parameter, which proved promising as a prognostic indicator.

Estimating the number of motor units was explored using the MUNE and MUNIX techniques. Felice et al., 1995 [29] demonstrated the reduction of MUNE in ALS, with greater amplitude of unitary potentials due to compensatory reinnervation. Baumann et al., 2012 [36] applied a Bayesian model to describe the loss of motor units over time, varying according to the clinical subtype. Neuwirth et al., 2017 [38] observed decline rates of up to 5.6% per month, while Boekestein et al., 2012 [35] and Ahn et al., 2010 [39] confirmed the high reproducibility and reliability of MUNIX.

Analysis of the complexity of sEMG signals has proved useful in detecting early alterations. Zhou et al., 2011 [41] characterized spontaneous patterns based on approximate entropy (ApEn), showing greater regularity in the discharge of motor units in ALS patients. Zhang et al., 2014 [45] observed that multiscale entropy (MSE) is sensitive to changes specific to ALS, identifying less complexity at smaller scales. Saidane et al., 2021 [55] proposed an effective classification system between different types of ALS and controls, achieving up to 99.8% accuracy.

Advanced processing techniques, such as the APFP (Automatic Progressive FastICA Peel-Off) algorithm proposed by Chen et al., 2018 [42], and the use of morphological attributes extracted by Antunes et al., 2023 [53], have shown great accuracy in decomposing sEMG signals and classifying ALS patients. These approaches, combined with machine learning algorithms such as Support Vector Machine (SVM), KNN, and AdaBoost, have shown high performance in patient stratification and early assessment of neuromuscular degeneration.

## Discussion

This systematic review highlighted the growing application of sEMG as a neuromuscular assessment tool in individuals with ALS, bringing together a significant diversity of methodological approaches, clinical objectives, and methodological and analytical strategies. Despite the heterogeneity of assessment protocols, certain consistent patterns emerge, highlighting the relevance of sEMG in the functional characterization of ALS, differentiation of clinical subtypes, longitudinal monitoring, and its potential use as a diagnostic support tool.

sEMG has proved to be a valuable tool in understanding this pathophysiology, allowing non-invasive assessment of neuromuscular function and the identification of objective markers of disease progression [59].

Among the most recurrent findings is the study of muscle fatigue, with evidence that central mechanisms – and not just peripheral ones – play a predominant role in the functional decline of ALS patients. Studies have shown that, even in the presence of discrete myoelectric responses, there is progressive failure of cortical and bulbar activation during sustained isometric tasks [31,32], reinforcing the understanding that fatigue in ALS involves central recruitment failures.

Another aspect that was widely explored was the characterization of muscle fasciculations, which are considered to be non-invasive biomarkers of neuronal excitability in ALS. Studies have identified distinct patterns of interspike intervals (ISI), suggesting different physiological origins for these discharges: spontaneous axonal and spinal motoneuron-mediated firing [33,34]. The SPiQE system has proved effective in automatically detecting fasciculations in high-density signals (HD-sEMG), with high sensitivity and specificity [46]. In longitudinal follow-up, a pattern of increase followed by a decline in fasciculations was observed as the disease progressed, demonstrating a direct association with patient survival [48,49].

sEMG offers a non-invasive approach to assessing neuromuscular function and is useful in functional characterization, monitoring disease progression, diagnostic support, and prognosis. Techniques for estimating the number of motor units, such as MUNE and MUNIX, have proved to be highly sensitive to the progression of ALS, with significant monthly declines in MUNIX greater than the changes observed in CMAP values and clinical scales [29,36,38]. The inter-observer reliability of the measurements has also been documented, expanding their potential for standardized clinical use [39].

Complexity analysis and the decomposition of sEMG signals have been areas of notable methodological innovation. Studies have applied multiscale entropy techniques, approximate entropy, wavelet transforms, cepstrum, and detrended fluctuation analysis (DFA) to discriminate signals from patients and controls [41,45]. In addition, supervised classifiers such as Support Vector Machine (SVM), K-Nearest Neighbors (KNN), Artificial Neural Network (ANN), and Convolutional Neural Network (CNN) were trained with attributes from the time and frequency domains, achieving high accuracy in distinguishing between patients with predominant upper and lower motor neuron involvement and healthy individuals [55].

The integration of sEMG with other assessment technologies was rare, although promising. Studies that have combined sEMG with magnetic resonance spectroscopy, MUS, and transcranial magnetic stimulation illustrate broader and potentially more sensitive approaches to the complexity of motor degeneration in ALS [31,51,54]. The use of portable and wireless systems was described, representing a promising trend for the future of home monitoring and decentralized clinical applications, enabling greater accessibility, patient comfort, and greater autonomy in the continuous collection of data in non-hospital environments.

Important methodological limitations have been identified, especially the heterogeneity of the sEMG signal acquisition and analysis protocols. Although established guidelines for sEMG acquisition and reporting exist, such as SENIAM and the more recent CEDE recommendations, their adoption across the included studies was only partial. In most cases, electrode placement followed SENIAM anatomical recommendations or similar standardized references. However, other methodological aspects including acquisition protocols, contraction tasks, rest intervals, and signal-processing procedures varied substantially between studies. These procedures were generally described by the authors, allowing reproducibility, but the lack of consistent methodological frameworks may contribute to the heterogeneity observed across studies. This finding highlights the importance of broader adoption of comprehensive guidelines, such as CEDE, to improve comparability and reproducibility in future sEMG research involving ALS.

Furthermore, the predominance of studies focused on upper and lower limbs, with an emphasis on distal muscles, represents a gap in the functional assessment of regions such as the torso, cervical, head, and neck. Future research should prioritize the standardization of sEMG signal acquisition protocols, allowing for greater comparability between studies and large-scale clinical validation. The investigation of the torso, cervical, head, and neck muscles should be encouraged, especially in subtypes of ALS with non-classical involvement. In addition, there is a need to develop open databases, clinically validated automated analysis tools, and longitudinal studies that use sEMG as a marker of response to experimental therapies. The combination of sEMG with other technological modalities can enrich the clinical and pathophysiological understanding of ALS and contribute to the development of new therapeutic approaches. Furthermore, the development and adoption of portable and wireless systems is recommended, with a view to application in home and rehabilitation contexts.

## Conclusion

This systematic review shows that sEMG has meaningful clinical potential for the neuromuscular assessment of individuals with ALS, particularly for quantifying muscle fatigue, characterizing fasciculation patterns, estimating the number of motor units (MUNE/MUNIX), and analyzing signal complexity. These parameters demonstrate sensitivity to disease progression and may contribute to improved diagnostic and prognostic accuracy.

However, despite these promising findings, the relatively small number of studies identified over nearly three decades indicates that sEMG remains an underexplored tool in ALS research. The marked heterogeneity in acquisition protocols, electrode configurations, and analytical methods further limits cross-study comparability and hinders the development of standardized clinical applications.

Future research should focus on protocol standardization, expansion of assessments to underrepresented muscle regions (e.g., bulbar, cervical, and trunk muscles), and the clinical validation of advanced analytical techniques, including machine learning. Greater integration of sEMG with other neurophysiological modalities and expanded use of portable and wireless systems may support more comprehensive and accessible monitoring approaches, ultimately strengthening the role of sEMG in the clinical management of ALS.

## Supporting information

S1 Table Motor clinical parameters evaluated in the included studies. Motor clinical parameters evaluated across the included studies involving individuals with amyotrophic lateral sclerosis (ALS), including electrophysiological, functional, and neuromuscular measures.
(DOCX)

S2 Table Participant characteristics. Demographic and clinical characteristics of participants included in the reviewed studies, including sample size, age, symptom duration, diagnostic criteria, and control group characteristics.
(DOCX)

S3 Table Muscles or muscle groups evaluated in the included studies. Muscles and muscle groups assessed by surface electromyography (sEMG) in the included studies involving individuals with ALS.
(DOCX)

S4 Table Data collection activities and rest/collection time in the included studies. Data collection protocols adopted in the included studies, including experimental activities, contraction tasks, rest periods, and recording duration during sEMG acquisition.
(DOCX)

S5 Table sEMG device characteristics. Technical characteristics of the sEMG devices used in the included studies, including electrode configuration, portability, amplifier specifications, and sampling frequency.
(DOCX)

S6 Table Signal processing features in surface sEMG studies. Signal processing methods applied to surface electromyography data in ALS studies, including filters, extracted features, software environments, and analysis techniques.
(DOCX)

S7 Table Summary of clinical data and sEMG signal processing outcomes in ALS studies. Summary of the main clinical findings, sEMG signal processing outcomes, and study observations reported in the included ALS studies.
(DOCX)

S1 Checklist PRISMA Checklist. PRISMA 2020 checklist describing the reporting items included in this systematic review.
(DOCX)
