# Supplementary material for: Applications of electromyography in Amyotrophic Lateral Sclerosis: A systematic review
Source: PLoS One. 2026 Jun 22;21(6):e0350029. doi: 10.1371/journal.pone.0350029 (PMC13286138; doi:10.1371/journal.pone.0350029)
Supplement: S1 Table — Motor clinical parameters evaluated across the included studies involving individuals with amyotrophic lateral sclerosis (ALS), including electrophysiological, functional, and neuromuscular measures. (DOCX) [file pone.0350029.s001.docx]

S1 Table. Motor Clinical Parameters Evaluated in the Included Studies.

| **Authors** | **Motor Clinical Parameters Evaluated** |
| --- | --- |
| Felice et al., 1995 | MUNE; CMAP; S-MUAP; Median nerve conduction; Muscle atrophy. |
| Baumann et al., 2012 | MUNE; MUAP; Motor unit half-life. |
| Bromberg et al., 1996 | MVIC; MUNE; CMAP; Muscle coactivation. |
| Neuwirth et al., 2017 | Muscle weakness. |
| van Dijk et al., 2010 | Motor neuron loss; Muscle weakness. |
| Kleine et al., 2008 | Fasciculations; Axonal and neuronal excitability. |
| Boekestein et al., 2012 | MUNE; CMAP; MRC; ALSFRS. |
| Nandedkar et al., 2022 | STEPIX; AMPIX; ALSFRS-R; MRC. |
| Neuwirth et al., 2010 | Fatigue; Fasciculations; Motor unit loss; Muscle weakness. |
| Ahn et al., 2010 | MUNE; Fatigue; Muscle weakness; ALSFRS-R; MRC. |
| Bashford et al., 2019 | FPs; FP morphology, frequency, and amplitude; Signal noise correlation. |
| Escorcio-Bezerra et al., 2016 | Fatigue; Fasciculations; Paresis/plegia; MUNIX; CMAP. |
| Kim et al., 2016 | MUNIX; CMAP; ALSFRS-R. |
| Antunes et al., 2023 | MUAP; EMG signal morphology changes. |
| Kent-Braun et al., 2000 | Central and peripheral fatigue; MVC; PCr depletion; Central and peripheral muscle activation via EMG and electrical stimulation. |
| Castro et al., 2023 | CutSP latency and duration; EMG amplitude suppression. |
| Zhang et al., 2014 | FPs; Hidden spontaneous muscle activity. |
| Saidane et al., 2021 | UMN and LMN characteristics; Gait duration; Muscle activation pattern during gait. |
| Jahanmiri-Nezhad et al., 2015 | Abnormal MUP propagation; Innervation zone dispersion; FPs. |
| Zhou et al., 2011 | Spontaneous motor unit activity; Action potential dispersion; Discharge complexity. |
| Alarcón-Jimenez et al., 2022 | Muscle coactivation; Fatigue; Muscle activation rhythm. |
| Weddell et al., 2021 | Motor unit firing rate; ISI; AHP; MFCV; MUAP area. |
| Sanjak et al., 2004 | Fatigue; Mechanical strength decline; EMG frequency spectrum changes. |
| Quintão et al., 2021 | UMN degeneration; intermuscular coherence (IMC). |
| Wannop et al., 2021 | Fasciculation frequency; RoCoFF; ADR. |
| Bashford et al., 2020a | Fasciculations; FF; MRC; MUNIX. |
| Bashford et al., 2020b | Fasciculation frequency and amplitude. |
| Nishikawa et al., 2022 | MU firing rate and variability; MU recruitment and derecruitment. |
| Planinc et al., 2023 | Fasciculation frequency; Electromechanical latency; Fasciculation depth, amplitude, and duration. |
| Kleine et al., 2012 | Fasciculations; F-wave; ISI; Fasciculation origin localization. |
| Noto et al., 2023 | IFR; Motor unit loss; Neuronal hyperexcitability. |
| Chen et al., 2018 | MU discharge; MUAP superposition. |
| Zhang et al., 2013 | Decreased MU recruitment; Abnormally amplified and prolonged MUAPs. |
| Zhou et al., 2012 | Presence of FPs. |

**Abbreviations**:

**ALSFRS**: Amyotrophic Lateral Sclerosis Functional Rating Scale; **ALSFRS-R**: Revised Amyotrophic Lateral Sclerosis Functional Rating Scale; **AMPIX**: Average Step Amplitude; **ADR**: Amplitude Dispersion Rate; **AHP**: Afterhyperpolarization Potential; **CMAP**: Compound Muscle Action Potential; **CutSP**: Cortical Silent Period; **EMG**: Electromyography; **FF**: Fasciculation Frequency; **FPs**: Fasciculation Potentials; **IFR**: Instantaneous Firing Rate; **ISI**: Interspike Interval; **MFCV**: Muscle Fiber Conduction Velocity; **MRC**: Muscle Strength (Medical Research Council Scale); **MU**: Motor Unit; **MUAP**: Motor Unit Action Potential; **MUNE**: Motor Unit Number Estimation; **MUNIX**: Motor Unit Number Index; **MVC**: Maximum Voluntary Contraction; **MVIC**: Maximum Voluntary Isometric Contraction; **PCr**: Phosphocreatine; **RoCoFF**: Rate of Change of Fasciculation Frequency; **S-MUAP**: Surface Motor Unit Action Potential; **STEPIX**: CMAP Step Number; **UMN**: Upper Motor Neuron; **LMN**: Lower Motor Neuron.

**Caption**:

Motor clinical parameters evaluated across the included studies involving individuals with ALS, including electrophysiological, functional, and neuromuscular measures.
