# Supplementary material for: Applications of electromyography in Amyotrophic Lateral Sclerosis: A systematic review
Source: PLoS One. 2026 Jun 22;21(6):e0350029. doi: 10.1371/journal.pone.0350029 (PMC13286138; doi:10.1371/journal.pone.0350029)
Supplement: S2 Table — Demographic and clinical characteristics of participants included in the reviewed studies, including sample size, age, symptom duration, diagnostic criteria, and control group characteristics. (DOCX) [file pone.0350029.s002.docx]

S2 Table. Participant Characteristics

| **Authors** | **Experimental Group (n)** | **Mean Age ± SD (Experimental)** | **Symptom Duration (months)** | **Diagnosis Time (months)** | **Patient Classification/Assessment** | **Control Group (n, Health)** | **Mean Age ± SD (Control)** |
| --- | --- | --- | --- | --- | --- | --- | --- |
| Felice et al., 1995 | 20 ALS | 61 (37–78) | Not Mentioned | Not Mentioned | Clinical and electrophysiological criteria | 16 Healthy | 39 (24–52) |
| Baumann et al., 2012 | 42 ALS | 58 ± 12 (29–78) | 616 days (112–3485) | Not Mentioned | Modified El Escorial criteria. Subgroups: Typical ALS, UMN-D, LMN-D. Motor unit half-life assessment. | 8 Healthy | Not Mentioned |
| Bromberg et al., 1996 | 10 ALS/MND | 56 (33–80) | Not Mentioned | Not Mentioned | El Escorial criteria. Muscle strength and electrophysiological evaluation. | Not Mentioned | Not Mentioned |
| Neuwirth et al., 2017 | 49 ALS | 59.3 ± 11.3 | 13.4 ± 5.8 | Not Mentioned | Revised El Escorial criteria. ALSFRS-R, MMT. | Not Mentioned | Not Mentioned |
| van Dijk et al., 2010 | 18 ALS | 64 (21–77) | 1.7 years (0.8–5.5) | Not Mentioned | El Escorial criteria. ALSFRS, MRC, Martin Vigorimeter. | 26 Healthy, age-matched | 61 (23–78) |
| Kleine et al., 2008 | 10 ALS | 62 (40–84) | 31 (6–60) | Not Mentioned | Revised El Escorial criteria. | None | Not Mentioned |
| Boekestein et al., 2012 | 18 ALS | 64 (21–77) | 20.4 (9.6–66) | Not Mentioned | Revised El Escorial criteria. MRC, ALSFRS. | 24 Healthy | 62 (49–78) |
| Nandedkar et al., 2022 | 26 ALS | 64.9 ± 9.6 | 17.9 ± 12.3 | Not Mentioned | ALSFRS-R, MRC. Classification: 1 possible, 7 probable, 18 definite. | 20 Healthy | 51 ± 15 |
| Neuwirth et al., 2010 | 7 ALS | 61 ± 12 (43–73) | ≤14 | 9.2 ± 2.6 | Revised El Escorial criteria (possible ALS). | 8 Healthy | 32 ± 8 (23–45) |
| Ahn et al., 2010 | 22 ALS | Not Mentioned | 5–48 | Not Mentioned | Revised El Escorial criteria. MRC, ALSFRS-R. | 62 Healthy | 20–70 |
| Bashford et al., 2019 | 6 ALS | 50–61 | 10–60 | Not Mentioned | Revised El Escorial criteria. MRC. | 2 (Benign fasciculation syndrome, MMN) | Not Mentioned |
| Escorcio-Bezerra et al., 2016 | 30 ALS | 62.3 ± 8.5 (50–83) | 19 ± 11 | Not Mentioned | Revised El Escorial criteria. | 51 Healthy | 42.3 ± 16.4 (24–75) |
| Kim et al., 2016 | 39 ALS | 60.1 ± 11.2 | 8.9 ± 7.8 | Not Mentioned | Revised El Escorial criteria. ALSFRS-R. | 40 Healthy | 59.3 ± 8.6 |
| Antunes et al., 2023 | 17 ALS (Spinal onset) | 59 ± 10 | ≤36 | Not Mentioned | Riluzole treatment. MRC > 3. | 24 Healthy | Not Mentioned |
| Kent-Braun et al., 2000 | 7 ALS | 50.3 ± 3.0 | Not Mentioned | Not Mentioned | World Federation of Neurology criteria. MRC, Ashworth, EMG. | 6 Healthy | 50.9 ± 3.2 |
| Castro et al., 2023 | 24 ALS | 59.5 | Not Mentioned | Not Mentioned | Awaji criteria. UMN dysfunction score. ALSFRS-R. | 28 (ADM), 13 (TA) | 63.5 (ADM), 57.0 (TA) |
| Zhang et al., 2014 | 9 ALS | 57 ± 7 | Not Mentioned | Not Mentioned | Definitive or Probable (with lab support) ALS. | 9 Healthy | 45 ± 16 |
| Saidane et al., 2021 | 31 ALS | Not Mentioned | Not Mentioned | Not Mentioned | Revised El Escorial criteria. UMNp: 14, LMNp: 17. | 14 Healthy | Not Mentioned |
| Jahanmiri-Nezhad et al., 2015 | 7 ALS | 56 ± 10 | Not Mentioned | Not Mentioned | El Escorial criteria. | 12 Healthy | 43 ± 18 |
| Zhou et al., 2011 | 9 ALS | 57 ± 7 | Not Mentioned | Not Mentioned | El Escorial criteria. EMG. | 9 Healthy | 45 ± 16 |
| Alarcón-Jimenez et al., 2022 | 23 ALS | 59 | >6 | 24 | El Escorial criteria. | Not Mentioned | Not Mentioned |
| Weddell et al., 2021 | 20 ALS | 63 (IQR 57–71) | Not Mentioned | ≤24 | Revised El Escorial criteria. | 5 (Benign fasciculation) | 38 |
| Sanjak et al., 2004 | 13 ALS | 50.5 ± 11.6 | Not Mentioned | Not Mentioned | ALS diagnosis (no specific scale). | 13 Healthy | 55.0 ± 17.8 |
| Quintão et al., 2021 | 13 ALS | 59.0 ± 9.0 | ≤36 | Not Mentioned | MRC, ALS criteria. | 20 Healthy | 47.0 ± 7.0 |
| Wannop et al., 2021 | 31 ALS | 60.5 (55.2–65.4) | 23.0 (IQR 19.5–28.5) | 13.2 (7.6–19.8) | ENCALS survival tool, FVC, ALSFRS-R. | Not Mentioned | Not Mentioned |
| Bashford et al., 2020a | 20 ALS | 64 | ≤24 | Not Mentioned | ALSFRS-R, MRC, revised El Escorial. | 5 (Benign fasciculation) | 42 |
| Bashford et al., 2020b | 10 ALS | 63 | 30 | Not Mentioned | ALSFRS-R, MRC, revised El Escorial. | Not Mentioned | Not Mentioned |
| Nishikawa et al., 2022 | 16 ALS | 65.4 ± 10.3 | Not Mentioned | 11.4 ± 4.4 | Awaji criteria, ALSFRS-R. | 16 Healthy | 60.1 ± 3.6 |
| Planinc et al., 2023 | 12 ALS | 57 (IQR 50–65) | 28 (IQR 17–49) | Not Mentioned | Revised El Escorial, ALSFRS-R, MRC. | 13 Healthy | 54 (40.5–64.5) |
| Kleine et al., 2012 | 7 ALS | 66 (57–74) | 33 (6–60) | Not Mentioned | Revised El Escorial and Awaji criteria. | 7 (Benign fasciculation) | 52 (37–71) |
| Noto et al., 2023 | 19 ALS | 65.0 ± 13.1 | 10 (median) | Not Mentioned | ALSFRS-R. Needle EMG of vastus lateralis. | 20 Healthy | 71.4 ± 6.6 |
| Chen et al., 2018 | 9 ALS | Not Mentioned | Not Mentioned | Not Mentioned | Revised El Escorial criteria. | Not Mentioned | Not Mentioned |
| Zhang et al., 2013 | 10 ALS | 57.3 ± 8.6 (48–75) | Not Mentioned | Not Mentioned | El Escorial (Probable or Definite). | 11 Healthy | 42.0 ± 17.4 (22–71) |
| Zhou et al., 2012 | 11 ALS | 57.4 ± 8.2 | Not Mentioned | Not Mentioned | El Escorial criteria. | Not Mentioned | Not Mentioned |

**Abbreviations:**

**ADM**: Abductor Pollicis Brevis; **ALS**: Amyotrophic Lateral Sclerosis; **ALSFRS**: Amyotrophic Lateral Sclerosis Functional Rating Scale; **ALSFRS-R**: Amyotrophic Lateral Sclerosis Functional Rating Scale – Revised; **EMG**: Electromyography; **ENCALS**: European Network to Cure ALS; **FVC**: Forced Vital Capacity; **IQR**: Interquartile Range; **LMN**: Lower Motor Neuron; **LMN-D**: Lower Motor Neuron Dominant; **MMN**: Multifocal Motor Neuropathy; **MMT**: Manual Muscle Testing; **MRC**: Medical Research Council; **SD**: Standard Deviation; **TA**: Tibialis Anterior; **UMN**: Upper Motor Neuron; **UMN-D**: Upper Motor Neuron Dominant.

**Caption**:

Demographic and clinical characteristics of participants included in the reviewed studies, including sample size, age, symptom duration, diagnostic criteria, and control group characteristics.
