# Supplementary material for: Applications of electromyography in Amyotrophic Lateral Sclerosis: A systematic review
Source: PLoS One. 2026 Jun 22;21(6):e0350029. doi: 10.1371/journal.pone.0350029 (PMC13286138; doi:10.1371/journal.pone.0350029)
Supplement: S3 Table — Muscles and muscle groups assessed by surface electromyography (sEMG) in the included studies involving individuals with ALS. (DOCX) [file pone.0350029.s003.docx]

S3 Table. Muscles or muscle groups evaluated in the included studies

| **Authors** | **Muscles / Muscle Groups** |
| --- | --- |
| Felice et al., 1995 | Thenar muscles |
| Baumann et al., 2012 | ADM (ulnar nerve); APB (median nerve); EDB (fibular nerve) |
| Bromberg et al., 1996 | Elbow flexors; Thenar and hypothenar muscles |
| Neuwirth et al., 2017 | APB; ADM; BB; TA; EDB; AH |
| van Dijk et al., 2010 | APB; OP; FPB; First and second lumbricals |
| Kleine et al., 2008 | BB (7 patients); VL (3 patients) |
| Boekestein et al., 2012 | Thenar muscle of the less affected hand (ALS patients) or nondominant hand (controls) |
| Nandedkar et al., 2022 | APB |
| Neuwirth et al., 2010 | APB; ADM; AHB; EDB (bilaterally) |
| Ahn et al., 2010 | ADM |
| Bashford et al., 2019 | BB; GM (bilaterally) |
| Escorcio-Bezerra et al., 2016 | TA; APB; ADM |
| Kim et al., 2016 | APB; FDI; ADM |
| Antunes et al., 2023 | FDI (right hand); EDC (both forearms) |
| Kent-Braun et al., 2000 | TA |
| Castro et al., 2023 | APB; TA |
| Zhang et al., 2014 | Thenar group |
| Saidane et al., 2021 | Right TA |
| Jahanmiri-Nezhad et al., 2015 | BB |
| Zhou et al., 2011 | Bilateral thenar muscles (APB) |
| Alarcón-Jimenez et al., 2022 | BB; TR; RF; TA |
| Weddell et al., 2021 | Bilateral BB |
| Sanjak et al., 2004 | BB; TA |
| Quintão et al., 2021 | FDI; EDC |
| Wannop et al., 2021 | BB (24 bilateral, 7 unilateral) |
| Bashford et al., 2020a | BB; Bilateral GM; APB; BB; EDB |
| Bashford et al., 2020b | BB; GM (symptom side) |
| Nishikawa et al., 2022 | VL (weaker side in ALS); VL (dominant side in controls) |
| Planinc et al., 2023 | Right BB; Right GM |
| Kleine et al., 2012 | GM |
| Noto et al., 2023 | Right VL |
| Chen et al., 2018 | FDI |
| Zhang et al., 2013 | Thenar muscles; FDI |
| Zhou et al., 2012 | FDI; Thenar muscle; BB |

**Abbreviations**

**ADM**: Abductor Digiti Minimi; **AH**: Abductor Hallucis; **AHB**: Abductor Hallucis Brevis; **APB**: Abductor Pollicis Brevis; **BB**: Biceps Brachii; **EDB**: Extensor Digitorum Brevis; **EDC**: Extensor Digitorum Communis; **FDI**: First Dorsal Interosseous; **FPB**: Flexor Pollicis Brevis; **GM**: Medial Gastrocnemius; **OP**: Opponens Pollicis; **RF**: Rectus Femoris; **TA**: Tibialis Anterior; **TR**: Triceps Brachii; **VL**: Vastus Lateralis.

**Caption**:

Muscles and muscle groups assessed by sEMG in the included studies involving individuals with ALS.
