# Supplementary material for: Applications of electromyography in Amyotrophic Lateral Sclerosis: A systematic review
Source: PLoS One. 2026 Jun 22;21(6):e0350029. doi: 10.1371/journal.pone.0350029 (PMC13286138; doi:10.1371/journal.pone.0350029)
Supplement: S4 Table — Data collection protocols adopted in the included studies, including experimental activities, contraction tasks, rest periods, and recording duration during sEMG acquisition. (DOCX) [file pone.0350029.s004.docx]

S4 Table. Data collection activities and rest/collection time in the included studies.

| **Authors** | **Activity During Collection** | **Collection/Rest Time** |
| --- | --- | --- |
| Felice et al., 1995 | Electrical stimulation of the median nerve at multiple points | Not Mentioned |
| Baumann et al., 2012 | Electrical stimulation of nerves and CMAP recording | At least 1000 stimuli per curve |
| Bromberg et al., 1996 | MVIC of handgrip and pinch; CMAP and MUNE using multipoint stimulation | Not Mentioned |
| Neuwirth et al., 2017 | Voluntary isometric contraction | <5 minutes per muscle |
| van Dijk et al., 2010 | Voluntary contraction for MUPs | Not specified |
| Kleine et al., 2008 | Rest with visual feedback to ensure relaxation; excluded contraction periods | 15 minutes |
| Boekestein et al., 2012 | Voluntary contraction of thenar muscle at five force levels; median nerve stimulation | Not specified |
| Nandedkar et al., 2022 | Median nerve stimulation; limb warming; EMG setup; CMAP scan protocol | ~10 minutes per exam; repeated after 30 minutes or 7 months |
| Neuwirth et al., 2010 | Voluntary resistance contraction from minimal to maximal effort | 30–45 minutes total |
| Ahn et al., 2010 | Supramaximal stimulation of ulnar nerve; isometric contraction of ADM | MUNIX <5 minutes per muscle |
| Bashford et al., 2019 | Rest; surface EMG without voluntary contraction | 30 minutes per muscle; ≥1 month between sessions |
| Escorcio-Bezerra et al., 2016 | Progressive isometric contractions recorded by surface EMG | 30 minutes to 6 hours between sessions |
| Kim et al., 2016 | Isometric contractions at five force levels | Not Mentioned |
| Antunes et al., 2023 | Coordinated finger elevation with isometric hold and relaxation | 6 minutes or until fatigue |
| Kent-Braun et al., 2000 | Intermittent isometric ankle dorsiflexion | 25 minutes total: 15 min at 30% MVC, 10 min at 50% MVC |
| Castro et al., 2023 | Isometric contraction with audio biofeedback; electrical sensory stimulation | 50% max force; irregular stimuli to avoid fatigue |
| Zhang et al., 2014 | Rest only | 3 minutes |
| Saidane et al., 2021 | Barefoot 5-meter walking with visual cue | 80 trials per participant |
| Jahanmiri-Nezhad et al., 2015 | Light voluntary contraction; passive FP recording at rest | 10–30 minutes |
| Zhou et al., 2011 | Full rest to record spontaneous activity | 4 minutes |
| Alarcón-Jimenez et al., 2022 | Maximal isometric contractions | 210 s rest + 5 × 1 s contractions with 5 s rest |
| Weddell et al., 2021 | Rest and light contraction with feedback | 30 min rest + 1 min light activity |
| Sanjak et al., 2004 | Maximal voluntary isometric contraction for 30 s | 30 s contraction; ≥5 min rest between attempts |
| Quintão et al., 2021 | Index finger contraction | 3 s contraction + 3 s rest × 6 min |
| Wannop et al., 2021 | Resting with pronated forearms, elbow 90–120° | 30 minutes |
| Bashford et al., 2020a | Rest; no voluntary activity | 30 minutes |
| Bashford et al., 2020b | Relaxed supine position on exam table | 3 × 30 minutes (9:00, 12:00, 15:00) |
| Nishikawa et al., 2022 | 30% MVC knee extension: ramp-up, hold, and relax | 15 s ramp-up + 15 s hold + 15 s relax |
| Planinc et al., 2023 | Supine rest | 30 minutes |
| Kleine et al., 2012 | Seated rest | 15 minutes |
| Noto et al., 2023 | Knee extension: 0–30% MVC ramp in 15 s; 10% MVC hold for 60 s | 60 seconds at 10% MVC |
| Chen et al., 2018 | Low-force isometric contraction followed by increased intensity | ≥5 seconds per contraction with adequate rest |
| Zhang et al., 2013 | Isometric contraction (FDI abduction, thumb flexion for thenar) | ≥2 seconds per level; sufficient rest |
| Zhou et al., 2012 | Full rest during signal recording | ≥3 minutes |

**Abbreviations**

**ADM**: Abductor Digiti Minimi; **ALSFRS**: Amyotrophic Lateral Sclerosis Functional Rating Scale; **ALSFRS-R**: Revised Amyotrophic Lateral Sclerosis Functional Rating Scale; **CMAP**: Compound Muscle Action Potential; **EMG**: Electromyography; **FPs**: Fasciculation Potentials; **MUNE**: Motor Unit Number Estimation; **MUNIX**: Motor Unit Number Index; **MVC**: Maximum Voluntary Contraction; **MVIC**: Maximum Voluntary Isometric Contraction; **MUPs**: Motor Unit Potentials.

**Caption**:

Data collection protocols adopted in the included studies, including experimental activities, contraction tasks, rest periods, and recording duration during sEMG acquisition.
