# Supplementary material for: Applications of electromyography in Amyotrophic Lateral Sclerosis: A systematic review
Source: PLoS One. 2026 Jun 22;21(6):e0350029. doi: 10.1371/journal.pone.0350029 (PMC13286138; doi:10.1371/journal.pone.0350029)
Supplement: S5 Table — Technical characteristics of the sEMG devices used in the included studies, including electrode configuration, portability, amplifier specifications, and sampling frequency. (DOCX) [file pone.0350029.s005.docx]

S5 Table. sEMG device characteristics.

| **Authors** | **Device Brand** | **Electrode Type** | **Portability** | **Amplifier** | **Sampling Frequency** |
| --- | --- | --- | --- | --- | --- |
| Felice et al., 1995 | Advantage EMG System | Self-Adhesive ECG Electrodes (1 × 3 Cm) | Not Mentioned | Not Mentioned | Not Mentioned |
| Baumann et al., 2012 | Nicolet Viking IV | Pre-Gelled Surface Electrodes (20 Mm Diameter) | Not Mentioned | Not Mentioned | Not Mentioned |
| Bromberg et al., 1996 | N/A | Surface Electrodes | Not Mentioned | Not Mentioned | Not Mentioned |
| Neuwirth et al., 2017 | Natus Medical Inc. | Disposable Adhesive Surface Electrodes (15 Mm) | Fixed, Clinical Setting | Keypoint™-Classic, Keypoint™.Net, Synergy™ | Not Mentioned |
| Van Dijk et al., 2010 | Biosemi | High-Density Electrode Grid (8 × 15 Ag–AgCl, 4 Mm Spacing) | Fixed, Clinical Setting | 130-Channel Amplifier (Active-One) | 2048 Hz |
| Kleine et al., 2008 | N/A | High-Density Surface Matrix (10 × 13 Electrodes, 5 Mm Interelectrode Distance) | Not Mentioned | Not Mentioned | 2000 Hz |
| Boekestein et al., 2012 | Biosemi | High-Density Grid (8 × 15 Ag–AgCl Electrodes, 4 Mm Spacing) | Not Mentioned | ActiveOne | 2048 Hz |
| Nandedkar et al., 2022 | Synergy EMG System | Disposable Surface Electrodes (Part #019-415200) | Not Mentioned | Not Mentioned | Not Mentioned |
| Neuwirth et al., 2010 | Medtronic | Adhesive Surface Electrodes (Ambu Blue Sensor Tab, 22 × 24 Mm) | Fixed, Clinical Setting | Keypoint Classic | Not Mentioned |
| Ahn et al., 2010 | Oxford Instruments | Flat Disk Surface Electrodes (10 Mm) | Not Mentioned | Synergy | Not Mentioned |
| Bashford et al., 2019 | TMS International BV | 64 Circular Electrodes (8 × 8 Grid; 4.5 Mm Diameter, 8.5 Mm Spacing) | Not Mentioned | Refa-64 EMG | 2048 Hz |
| Escorcio-Bezerra et al., 2016 | Neurosoft | Disposable Pre-Gelled Surface Electrodes (10 × 30 Mm) | Fixed, Clinical Setting | Not Mentioned | 5–10000 Hz |
| Kim et al., 2016 | Oxford Instruments | Flat Disk Surface Electrodes (10 Mm) | Fixed, Clinical Setting | Synergy | Not Mentioned |
| Antunes et al., 2023 | Biosignalsplux | Surface (sEMG) | Portable Biomedical Device | Not Mentioned | 1000 Hz |
| Kent-Braun et al., 2000 | N/A | Surface Electrodes (10 Mm Diameter) | Not Mentioned | Not Mentioned | Not Mentioned |
| Castro et al., 2023 | Natus Inc | Surface | Not Mentioned | Not Mentioned | 3000 Hz |
| Zhang et al., 2014 | TMS International BV | Flexible Matrix With 64 Sensors (8 × 8) | Not Mentioned | Refa EMG System | 2000 Hz |
| Saidane et al., 2021 | N/A | N/A | Not Mentioned | Not Mentioned | Not Mentioned |
| Jahanmiri-Nezhad et al., 2015 | TMS International BV | Linear Surface Electrode Array (20 Channels) | Fixed, Clinical Setting | Refa128 EMG System | 2000 Hz |
| Zhou et al., 2011 | TMS International BV | Flexible Surface Matrix (8 × 8; 64 Electrodes, 1.2 Mm, 4 Mm Spacing) | Fixed, Clinical Setting | Refa128 Advanced EMG/EEG System | 2000 Hz |
| Alarcón-Jimenez et al., 2022 | BTS FreeEmg | Lessa Pediatric Electrodes (30 Mm Diameter) | Wireless (Bluetooth) | Not Mentioned | 1000 Hz |
| Weddell et al., 2021 | TMS International BV | 64 Circular Electrodes (8 × 8 Grid; 4.5 Mm Diameter, 8.5 Mm Spacing) | Fixed, Clinical Setting | Refa-64 EMG | 2048 Hz |
| Sanjak et al., 2004 | Custom Preamp | Surface Electrodes (10 Mm Diameter, 35 Mm Spacing) | Not Mentioned | A/D Conversion With Data Translation DT2801 | 500 Hz |
| Quintão et al., 2021 | BioPlux (8 Analog Channels) | Surface | Not Mentioned | Not Mentioned | 1000 Hz |
| Wannop et al., 2021 | TMS International BV | HDSEMG (64 Circular Electrodes, 8 × 8) | Not Mentioned | Refa-64 | Not Mentioned |
| Bashford et al., 2020a | TMS International BV | 64 Circular Surface Electrodes (8 × 8, 4.5 Mm Diameter, 8.5 Mm Spacing) | Not Mentioned | Wired | 2048 Hz |
| Bashford et al., 2020b | TMS International BV | HDSEMG – 64 Circular Electrodes (8 × 8, 4.5 Mm, 8.5 Mm Spacing) | Not Mentioned | Refa-64 EMG | 2048 Hz |
| Nishikawa et al., 2022 | OT Bioelettronica | HDSEMG – 64 Multiple Electrodes (1 Mm Diameter, 8 Mm Spacing) | Not Mentioned | 16-Bit A/D Converter (Quattrocento, OT Bioelettronica) | 2048 Hz |
| Planinc et al., 2023 | TMS International BV | 64 Circular Electrodes (8 × 8 Grid; 4.5 Mm, 8.5 Mm Spacing) | Not Mentioned | Refa-64 EMG Amplifier | 2048 Hz |
| Kleine et al., 2012 | Biosemi | HD-sEMG Matrix (10 × 13 Electrodes) | Not Mentioned | Not Mentioned | 2048 Hz |
| Noto et al., 2023 | OT Bioelettronica | Semi-Disposable Adhesive Sheet With 64 Channels (13 × 5, 1 Mm, 8 Mm) | Not Mentioned | Not Mentioned | 2048 Hz |
| Chen et al., 2018 | TMS International BV | HDSEMG – 64 Channels (8 × 8, 1.2 Mm Diameter, 4 Mm Spacing) | Not Mentioned | Refa128 Amplifier | 2000 Hz |
| Zhang et al., 2013 | TMS International BV | Flexible Matrix With 64 Channels (8 × 8) | Not Mentioned | Refa128 EMG System, 60 dB Gain | 2000 Hz |
| Zhou et al., 2012 | TMS International BV | Flexible Matrix for Thenar and FDI; Linear Bar (20 Electrodes for BB) | Not Mentioned | Refa128 EMG System | 2000 Hz |

**Abbreviations**

**ALSFRS**: Amyotrophic Lateral Sclerosis Functional Rating Scale; **BB**: Biceps Brachii; **EMG**: Electromyography; **FDI**: First Dorsal Interosseous; **HDSEMG**: High-Density Surface Electromyography; **sEMG**: Surface Electromyography.

**Caption**:

Technical characteristics of the sEMG devices used in the included studies, including electrode configuration, portability, amplifier specifications, and sampling frequency.
