# Supplementary material for: Applications of electromyography in Amyotrophic Lateral Sclerosis: A systematic review
Source: PLoS One. 2026 Jun 22;21(6):e0350029. doi: 10.1371/journal.pone.0350029 (PMC13286138; doi:10.1371/journal.pone.0350029)
Supplement: S6 Table — Signal processing methods applied to surface electromyography data in ALS studies, including filters, extracted features, software environments, and analysis techniques. (DOCX) [file pone.0350029.s006.docx]

S6 Table. Signal processing features in surface sEMG studies.

| **Authors** | **Filters** | **Signal Features** | **Software / Processing Language** | **Analysis Technique** |
| --- | --- | --- | --- | --- |
| **Felice et al., 1995** | Standard configuration of Advantage EMG System | Negative peak area (n-p) of CMAP and S-MUAPs; mean of action potentials | Advantage EMG System (proprietary software) | Multipoint stimulation technique; estimated MUNE from CMAP/S-MUAPs |
| **Baumann et al., 2012** | Not Mentioned | CMAP and MUAP size | Modified Nicolet Biomedical software | Bayesian method for MUNE; exponential decay model |
| **Bromberg et al., 1996** | Not Mentioned | CMAP amplitude and negative peak area | Not reported | Multipoint stimulation for MUNE |
| **Neuwirth et al., 2017** | Not Mentioned | MUNIX from CMAP area and power | R (R Core Team, 2012) | Mathematical model for MUNIX estimation |
| **van Dijk et al., 2010** | 0.16–400 Hz band-pass | CMAP and individual MUPs; MUNE ratio | SPSS | Spatio-temporal MUP identification |
| **Kleine et al., 2008** | 3–400 Hz; high-pass 15 Hz | ISI histograms; fasciculation discharge patterns | Not specified | HD-EMG decomposition based on spatiotemporal patterns |
| **Boekestein et al., 2012** | 0.16–400 Hz | Interference patterns; MUNIX; HD-MUNE; EMG area and power | MATLAB 7.10 | MUNIX and HD-MUNE estimation; regression and variance analysis |
| **Nandedkar et al., 2022** | Not Mentioned | CMAP scan for MUNE; motor unit loss and reinnervation | Excel; MFitScan | STEPIX and AMPIX indexes development; logarithmic regression; iterative modeling |
| **Neuwirth et al., 2010** | High-pass: 2 Hz; Low-pass: 10000 Hz | CMAP area and power; MUNIX calculation | Not Mentioned | MUNIX based on ICMUC (CMAP power / area) / (SIP power / area) |
| **Ahn et al., 2010** | Not Mentioned | CMAP and SIP power/area | DOS-based MUNIX software | ICMUC = (CMAP power / area) / (SIP power / area); regression model |
| **Bashford et al., 2019** | 20–500 Hz; noisy channel removal | Fasciculation potential detection; signal amplitude and morphology | MATLAB (R2014a) | Creation of a “super-channel” by combining signals from multiple electrodes; linear model based on the relationship between noise and fasciculation potential. |
| **Escorcio-Bezerra et al., 2016** | 5–10000 Hz | CMAP amplitude and SIP | Excel spreadsheet | Power regression MUNIX; Nandedkar’s model |
| **Kim et al., 2016** | Not Mentioned | CMAP and SIP; MUNIX and CMAP indexes | DOS-based software | SIMUNIX, SICMAP equations; MUNIX & CMAP index formula |
| **Antunes et al., 2023** | Analog: 25–450 Hz; Digital Butterworth: 10–300 Hz | Amplitude, zero-crossing, waveform length; spectral entropy; wavelet/fractal features | Python (TSFEL Library) | AdaBoost with Random Forest; hyperparameter tuning |
| **Kent-Braun et al., 2000** | Not Mentioned | CMAP peak amplitude and negative wave duration | NMR1 (New Methods Research) | Pre/post-exercise comparison |
| **Castro et al., 2023** | 30 Hz – 10000 Hz | CutSP onset latency and duration | MATLAB R2018a | Correlation between latency time and UMN dysfunction score. |
| **Zhang et al., 2014** | 5th-order Butterworth: 20–500 Hz | PSD analysis; multiscale entropy (MSE) | Spike2; SPSS v16 | Spectral and complexity analysis |
| **Saidane et al., 2021** | Not Mentioned | MAV, VAR, Skewness, ZCR, RMS, WAMP, Hjorth mobility/complexity, SampEn; MDF; STFT Cepstrum; DWT | Not Mentioned | KNN; SVM; LDA; ANN; CNN |
| **Jahanmiri-Nezhad et al., 2015** | 20–500 Hz | Manual MUP and FP extraction | MATLAB | Clustering and innervation zone dispersion/spread algorithms |
| **Zhou et al., 2011** | 20–500 Hz; 60 Hz notch | Spontaneous activity pattern classification | MATLAB; Spike2 | ApEn complexity; spike sorting; Approximate Entropy |
| **Alarcón-Jimenez et al., 2022** | 4th-order Butterworth: 20–400 Hz | RMS | MATLAB R2021a | Peak detection and analysis using *findpeaks*; smoothdata |
| **Weddell et al., 2021** | 10–500 Hz | ISI and firing patterns | Custom MATLAB scripts | Progressive FastICA decomposition/peel-off |
| **Sanjak et al., 2004** | 25–250 Hz | Force fatigue index (FFI); median frequency shift (MFS) | Visual Basic 6 | Cooley-Tukey FFT for MFS |
| **Quintão et al., 2021** | 3rd-order Butterworth: 10–500 Hz | DFA; Lempel-Ziv complexity; MSE; mean frequency | Python | Temporal and spectral features for ML models: Decision Trees, RF, AdaBoost, LDA, KNN |
| **Wannop et al., 2021** | Not Mentioned | Fasciculation frequency and amplitude dispersion | MATLAB (R2014a) | SPiQE fasciculation detection |
| **Bashford et al., 2020a** | Not Mentioned | Amplitude median and IQR; fasciculation frequency | MATLAB (R2014a) | SPiQE automated analysis |
| **Bashford et al., 2020b** | Not Mentioned | Fasciculation amplitude variation during the day | MATLAB (R2014a) | SPiQE-based detection; noise filtering; voluntary activity exclusion |
| **Nishikawa et al., 2022** | 10–500 Hz | Motor unit discharge rate (MUs) | MATLAB 2021b | Convolutive BSS |
| **Planinc et al., 2023** | 20–500 Hz; 50 Hz notch | Fasciculation amplitude and timing; frequency | MATLAB R2021a | SPiQE fasciculation detection |
| **Kleine et al., 2012** | 0.16–400 Hz | Fasciculation ISI histogram analysis | Not Mentioned | Potential template matching algorithms |
| **Noto et al., 2023** | 10–500 Hz | MU firing rate (IFR); IFR histograms | MATLAB R2018b | Kernel convolution decomposition; pattern matching algorithm |
| **Chen et al., 2018** | 10–500 Hz | MU discharge detection; matching rate (MR), FNR, FDR | MATLAB | Automatic Progressive FastICA Peel-Off (APFP) framework |
| **Zhang et al., 2013** | 20–500 Hz | EMG amplitude kurtosis; clustering index (CI) | MATLAB R2008a | LDA-based discrimination; regression |
| **Zhou et al., 2012** | 20–500 Hz | Fasciculation detection using thresholding | Spike2 v5.12; MATLAB R2008a | Cumulative FP interval distribution; Mills model |

**Abbreviations**

**ANN**: Artificial Neural Network; **APFP**: Automatic Progressive FastICA Peel-off; **ApEn**: Approximate Entropy; **BSS**: Blind Source Separation; **CMAP**: Compound Muscle Action Potential; **CNN**: Convolutional Neural Network; **DFA**: Detrended Fluctuation Analysis; **DWT**: Discrete Wavelet Transform; **FastICA**: Fast Independent Component Analysis; **FDR**: False Discovery Rate; **FNR**: False Negative Rate; **FP**: Fasciculation Potential; **FFT**: Fast Fourier Transform; **HD-EMG**: High-Density Electromyography; **HD-MUNE**: High-Density Motor Unit Number Estimation; **ICMUC**: Ideal Case Motor Unit Count; **IFR**: Instantaneous Firing Rate; **ISI**: Interspike Interval; **KNN**: K-Nearest Neighbors; **LDA**: Linear Discriminant Analysis; **LMN**: Lower Motor Neuron; **LZC**: Lempel-Ziv Complexity; **MAV**: Mean Absolute Value; **ML**: Machine Learning; **MR**: Matching Rate; **MSE**: Multiscale Entropy; **MUAP**: Motor Unit Action Potential; **MUNE**: Motor Unit Number Estimation; **MUNIX**: Motor Unit Number Index; **PSD**: Power Spectral Density; **RF**: Random Forest; **RMS**: Root Mean Square; **SampEn**: Sample Entropy; **SIP**: Surface Interference Pattern; **SPiQE**: Surface Potential Quantification Engine; **STFT**: Short-Time Fourier Transform; **SVM**: Support Vector Machine; **UMN**: Upper Motor Neuron; **VAR**: Variance; **WAMP**: Willison Amplitude; **ZCR**: Zero Crossing Rate.

**Caption**:

Signal processing methods applied to surface electromyography data in ALS studies, including filters, extracted features, software environments, and analysis techniques.
