# Supplementary material for: Applications of electromyography in Amyotrophic Lateral Sclerosis: A systematic review
Source: PLoS One. 2026 Jun 22;21(6):e0350029. doi: 10.1371/journal.pone.0350029 (PMC13286138; doi:10.1371/journal.pone.0350029)
Supplement: S7 Table — Summary of the main clinical findings, sEMG signal processing outcomes, and study observations reported in the included ALS studies. (DOCX) [file pone.0350029.s007.docx]

S7 Table. Summary of clinical data and sEMG signal processing outcomes in ALS studies.

| **Authors** | **Clinical Data Results** | **EMG Processing Method Results** | **Study Observations** |
| --- | --- | --- | --- |
| Felice et al., 1995 | ALS patients had significantly fewer MUs (MUNE) and larger S-MUAPs, suggesting collateral sprouting. | Multipoint stimulation (MPS) technique showed high reproducibility (test-retest r = 0.99 for ALS). | Validated MPS for tracking disease progression; potential clinical trial outcome measure. |
| Baumann et al., 2012 | MU lifespan varied by ALS type; typical ALS had shortest lifespan. | Bayesian model estimated MU number/size and fit exponential decay pattern. | Faster MU loss correlated with lower survival; phenotypic insights provided. |
| Bromberg et al., 1996 | High test-retest reliability for MVIC and MUNE; coactivation observed. | Multipoint MUNE reproducible; CMAP variability affected correlation. | No intervention. |
| Neuwirth et al., 2017 | MUNIX, CMAP, ALSFRS-R declined monthly; MUNIX most sensitive pre-symptom. | MUNIX more sensitive than CMAP or ALSFRS-R; early biomarker potential. | No intervention. |
| van Dijk et al., 2010 | MUNE showed greatest decline over time compared to ALSFRS and CMAP. | High-density MUNE sensitive; differentiated progression speed. | No intervention. |
| Kleine et al., 2008 | Identified two fasciculation patterns: axonal and neuronal. | ISI-based HD-EMG distinguished these patterns effectively. | No intervention. |
| Boekestein et al., 2012 | MUNIX and HD-MUNE correlated; better sensitivity than CMAP or ALSFRS. | HD-MUNE had better reproducibility, MUNIX better stability. | No intervention. |
| Nandedkar et al., 2022 | STEPIX reduced, AMPIX increased; linked to denervation/reinnervation. | Strong correlation with MFitScan MUNE; low variability in STEPIX. | No intervention. |
| Neuwirth et al., 2010 | MUNIX scores lower in ALS; declined more than ALSFRS-R or SVC. | MUNIX correlated with CMAP, ALSFRS-R, and SVC; useful for monitoring. | No intervention. |
| Ahn et al., 2010 | Lower MUNIX in weaker muscles; no ALSFRS-R correlation. | High reproducibility (intra/inter-operator). | No intervention. |
| Bashford et al., 2019 | ALS fasciculations detected at high frequency (50–56/min). | Algorithm achieved 83.6% sensitivity, 91.6% specificity. | No intervention. |
| Escorcio-Bezerra et al., 2016 | MUNIX detected denervation before strength/CMAP loss. | High AUC (0.95) in ROC; 77% sensitivity to detect denervation. | No intervention. |
| Kim et al., 2016 | SIMUNIX, SICMAP lower in ALS; SIMUNIX correlated better with ALSFRS-R. | SIMUNIX more sensitive and specific than SICMAP (AUC = 0.95). | No intervention. |
| Antunes et al., 2023 | ML analysis of EMG features achieved 84% accuracy for ALS detection. | Morphological features improved signal interpretability. | No intervention. |
| Kent-Braun et al., 2000 | ALS showed central activation failure, less PCr depletion. | CMAP amplitude decreased; duration unchanged. | Stimulation revealed central fatigue not present in controls. |
| Castro et al., 2023 | CutSP latency increased in ALS; correlated with UMN dysfunction. | Reduced EMG suppression; logistic regression predicted UMN signs. | CutSP more sensitive than TMS. |
| Zhang et al., 2014 | ALS showed higher spectral power; complex MSE profile. | MSE more sensitive than PSD to detect ALS changes. | No intervention. |
| Saidane et al., 2021 | Activation patterns distinguished UMNp/LMNp/healthy groups. | ML classification accuracy up to 99.8% with PCA, SFS. | No intervention. |
| Jahanmiri-Nezhad et al., 2015 | ALS had increased IZ length and dispersion. | EMG showed abnormal propagation patterns. | No intervention. |
| Zhou et al., 2011 | Three firing patterns identified; ALS had lowest complexity. | ApEn used to differentiate spontaneous discharge patterns. | No intervention. |
| Alarcón-Jimenez et al., 2022 | ALS had increased coactivation; fatigue patterns observed. | Smoothed data highlighted muscle activation patterns. | Isometric contraction useful for analysis; may aid function. |
| Weddell et al., 2021 | ALS muscles had shorter ISIs, larger MUAPs. | HDSEMG with FastICA accurately identified 287 ALS units. | No intervention. |
| Sanjak et al., 2004 | ALS patients showed greater mechanical than myoelectric fatigue. | Median frequency analysis confirmed findings. | No intervention. |
| Quintão et al., 2021 | Differences in forearm EMG between ALS and controls. | DFA and peak frequency achieved 94% classification accuracy. | No intervention. |
| Wannop et al., 2021 | ALSFRS-R progression rate and survival linked to RoCoFF. | High RoCoFF associated with shorter survival. | No intervention. |
| Bashford et al., 2020a | ALS biceps showed fasciculation frequency shifts. | SPiQE algorithm detected over 900,000 fasciculations. | No intervention. |
| Bashford et al., 2020b | ALS patients had low awareness of fasciculations. | SPiQE algorithm maintained high reliability under noise. | No intervention. |
| Nishikawa et al., 2022 | ALS had elevated MU firing rates correlated with severity. | HDSEMG effective and non-invasive for MU analysis. | No intervention. |
| Planinc et al., 2023 | ALS had longer electromechanical latency, deeper fasciculations. | SPiQE showed accurate detection at depth (30 mm). | No intervention. |
| Kleine et al., 2012 | ALS fasciculations overlapped with benign types. | Template matching distinguished fasciculation types. | No intervention. |
| Noto et al., 2023 | ALS had increased MU firing during low-force contractions. | HDSEMG assessed hyperexcitability effectively. | No intervention. |
| Chen et al., 2018 | ALS EMG showed sparse signals from degeneration. | APFP decomposed signals automatically with 99.2% accuracy. | No intervention. |
| Zhang et al., 2013 | ALS MUAPs showed high amplitude/duration. | Combined analysis improved sensitivity to 90%, specificity to 100%. | No intervention. |
| Zhou et al., 2012 | Fasciculations detected in all muscles tested. | HDSEMG faster and more sensitive than needle EMG. | No intervention. |

**Abbreviations**

**ALS**: Amyotrophic Lateral Sclerosis; **ApEn**: Approximate Entropy; **CMAP**: Compound Muscle Action Potential; **CutSP**: Cutaneous Silent Period; **DFA**: Detrended Fluctuation Analysis; **EMG**: Electromyography; **HDSEMG**: High-Density Surface Electromyography; **IZ**: Innervation Zone; **MSE**: Multiscale Entropy; **MPS**: Multipoint Stimulation; **MUNIX**: Motor Unit Number Index; **MUNE**: Motor Unit Number Estimation; **MU**: Motor Unit; **MVIC**: Maximal Voluntary Isometric Contraction; **PCr**: Phosphocreatine; **PSD**: Power Spectral Density; **RoCoFF**: Rate of Change of Firing Frequency; **SPiQE**: Surface Potential Quantification Engine; **S-MUAP**: Single Motor Unit Action Potential; **SVC**: Slow Vital Capacity; **TMS**: Transcranial Magnetic Stimulation.

**Caption**:

Summary of the main clinical findings, sEMG signal processing outcomes, and study observations reported in the included ALS studies.
