# Supplementary material for: Applications of electromyography in Amyotrophic Lateral Sclerosis: A systematic review
Source: PLoS One. 2026 Jun 22;21(6):e0350029. doi: 10.1371/journal.pone.0350029 (PMC13286138; doi:10.1371/journal.pone.0350029)
Supplement: Attachment — Submitted filename: Response Letter.pdf [file pone.0350029.s011.pdf]

Dear Editor and Reviewers,

We would like to sincerely thank the reviewers for their careful reading of our manuscript and for their constructive comments and suggestion.

Thank you for this valuable suggestion. In response, we added a brief discussion addressing how the included studies relate to established sEMG guidelines, particularly the SENIAM and CEDE recommendations.

Although the assessment of compliance with these guidelines was not part of the predefined methodological criteria of this review, we performed a qualitative appraisal of the included studies. Most articles described electrode placement following SENIAM recommendations or other standardized anatomical references. However, other methodological aspects, such as acquisition protocols, task execution, rest periods, and signal-processing procedures, varied considerably across studies, despite being clearly reported by the authors.

This observation has now been incorporated into the Discussion section to highlight that, while electrode placement often follows recognized guidelines, substantial variability remains in other stages of sEMG acquisition and analysis. This reinforces the need for broader adoption of comprehensive standardization frameworks, such as CEDE, in future ALS research. The new discussion appears on **page 16, paragraph 4**.
