# Supplementary material for: Applications of electromyography in Amyotrophic Lateral Sclerosis: A systematic review
Source: PLoS One. 2026 Jun 22;21(6):e0350029. doi: 10.1371/journal.pone.0350029 (PMC13286138; doi:10.1371/journal.pone.0350029)
Supplement: Attachment — Submitted filename: Response Letter.docx [file pone.0350029.s010.docx]

Dear Reviewers,

We sincerely thank you for the time, attention, and thoughtful feedback provided during the evaluation of our manuscript. Your detailed comments greatly contributed to strengthening the clarity, rigor, and methodological consistency of this systematic review. Below, we present a point-by-point response to each comment, describing the revisions implemented and clarifying the decisions made during the review process.

1. The title does not accurately reflect the topic of the review and should be amended. Specifically, motion analysis is not a part of the review and should therefore not be mentioned.

*We appreciate the observation. The title has been revised as suggested to more accurately reflect the scope of the review.*

*Revised title: “Applications of Electromyography in Amyotrophic Lateral Sclerosis: A Systematic Review.”*

2. The introduction refers to difficulty in establishing standardised guidelines, but there is no mention in the manuscript about guidelines for sEMG use that have already been established. These should, at least be mentioned, and in the best case I think it would be valuable if the reported studies were assessed by these guidelines. Guidelines to consider are: SENIAM (<http://seniam.org/>, <https://pubmed.ncbi.nlm.nih.gov/11018445/> ) and CEDE (<https://www.sciencedirect.com/special-issue/10CFPDX6CN9> ). The lack of consideration of these standards reduces the value of the review, and misses an opportunity to highlight the availability of these standards to the ALS research and clinical community.

*We thank the reviewer for this observation. We revised the Introduction and added a dedicated paragraph on page 2, paragraph 6, explicitly addressing the SENIAM and CEDE guidelines and emphasizing their relevance for the standardization of electrode placement, acquisition parameters, and signal-processing procedures. We also discuss how the lack of adherence to these guidelines contributes to methodological heterogeneity and constitutes an important limitation among the included studies. This update strengthens the methodological context of the review and fully addresses the reviewer’s recommendation.*

3. The research questions would benefit from some refinement. Particularly, the term clinical is used a lot, but this does not always seem appropriate. For example, RQ2 asks what the clinical characteristics of the participants are reported. The information includes age, which is not specifically a clinical feature. Inter-related with this point (and see note on risk of bias below), other features such as sex characteristics are not included. What is the rationale for not including this?

Additionally, RQ5 asks about the ‘technical properties of sEMG’ – it seems it would be more appropriate that this stated ‘what hardware has been used for sEMG acquisition’ – or similar. RQ8 asks whether ‘biofeedback’ was used during ‘interventions’ and here interventions seems to reflect the completion of the study or experiment within an observational study design (i.e., the neuromuscular properties are being observed). I appreciate this second point is maybe more minor, but I think it is important in terms of ensuring correct and appropriate language is used throughout to improve clarity of the manuscript.

*The research questions were revised as suggested to improve terminological precision and clarity. We removed the term “clinical” where it was not appropriate, simplified the question regarding participant characteristics, reformulated RQ5 to explicitly address hardware and acquisition parameters, and adjusted RQ8 to avoid the incorrect use of the term “interventions” in observational study designs. We also refined the wording of the remaining questions to ensure greater objectivity and consistency with recommended terminology. All modifications are reflected in the updated Research Questions Table, now presented on page 4.*

4. It would be helpful to the reader to at least have a summary of the search strategy in this manuscript. Otherwise you are demanding people download an additional article to understand the work that has been conducted. I think it is better if this manuscript can standalone and be a self-contained piece of work.

*We thank the reviewer for this important suggestion. In response, we incorporated a concise summary of the search strategy directly into the Methods section, ensuring that the manuscript is self-contained and does not require consultation of external documents. These additions, which include the keywords and Boolean operators, are now presented on page 5. This revision improves the transparency and completeness of the manuscript.*

5. The assessment of methodological quality is described as part of the data analysis process in the methods section, but in the results section it appears to be stated that this was part of the selection process: “After eligibility assessment and methodological quality assessment, 34 studies were included in the quantitative analysis.” It should be clear in the methods section how this assessment was carried out and, if it was solely for the purpose of study selection, the inclusion/exclusion limits or thresholds should be explained.

We thank the reviewer for this observation. The Methods section has been revised to clarify that both the methodological quality assessment (GRADE) and the risk of bias assessment (PROBAST) were used as eligibility criteria. We now explicitly state that studies classified as having very low evidence by GRADE or that present a high risk of bias in any domain of PROBAST were excluded from the final synthesis. These clarifications have been added on page 7, in the Data Analysis section.

We are presenting below the image referring to the PROBAST of the included articles.

6. The methods describe a risk of bias assessment as having been completed as part of data analysis, but the results of this assessment are not reported. The prisma checklist also states this was not done. As the aim of the review seems to be to highlight the value of sEMG in ALS neuromuscular assessment this seems important to have completed and be reported.

*We thank the reviewer for this important observation. We clarified in the Methods section that the risk of bias assessment using PROBAST was employed as an eligibility criterion, rather than as a post-selection analytical step. Only studies classified as having low risk of bias across all PROBAST domains were included in the final synthesis; therefore, no studies with high or unclear risk of bias were retained, which explains why no risk-of-bias results appeared in the original Results section. This information is now more clearly presented on page 7, in the Data Analysis section.*

7. It seemed to that much of the information in appendices could have been included in the main manuscript so the reader has fuller insight into the studies assessed. For example, could details in appendices 3, 4, and 8 not be presented as pie charts? Could the table in appendix 9 not be in the main text? Are there publisher guidelines which prevent more tables/figures being included?

*We thank the reviewer for this suggestion. In response, we have added figures to the main manuscript summarizing the muscles analyzed and the types of feedback/outcomes assessed, which condense information previously presented in the appendices and improve clarity for the reader. The remaining appendices were maintained to preserve detailed study-level information without overloading the main text.*

8. There are inconsistencies in the details presented across some of the tables in the appendices.

For example, appendix 6 – SpiQE is reported as being both an analysis technique and an algorithm for some entries and just as an analysis technique in others (with algorithm stated as ‘not mentioned’). This also led to me asking how have you defined ‘analysis technique’ and ‘algorithm’ – what is the distinction between them? In the manuscript text the software used is not reported, but it would be useful to have a sense of what has been used without having to download the appendix. Equally, it would also be valuable to know which of the algorithms are commercially available and which available open source.

Further example, appendix 5 – REFA is defined as both a brand and an amplifier. Where it is entered as a brand the statement under ‘amplifier’ is ‘not mentioned’.

In appendix 7 – why is the distinction between ‘other devices’ and ‘neuroimaging’ important? Is neuroimaging not simply another device? I ask this because stating ‘Not mentioned’ could be seen as a negative for the study, but in this case it reflects the fact that neuroimaging was not appropriate to address that study’s specific research question.

*We have revised all tables and appendices for inconsistencies. In regard to Appendix 6, our initial idea in having two columns, one for analysis technique and one for algorithms, was to provide more information about the processing pipeline without having too much information on a single column. However, we agree that the distinction between the two terms is not clear in the manuscript, and thus decided to join both columns into a single one: analysis technique. We have integrated the infformation from both columns accordingly.*

*In Appendix 5, REFA is the name of the device, while TMSi is the brand of the device. TMS International BV was rebranded as "TMSi — an Artinis company"; however, we chose to maintain the legacy name in the corresponding entries as they are reported as so in the collected manuscripts. We have corrected all inconsistencies related to these entries.*

*As for Appendix 7, the rationale behind this division was similar to that employed in Appendix 6, i.e., to have more granularity in the described data. As such, we have also joined both columns into a single one without loss of information.*

More minor points:
1. Text in the abstract could be rephrased to improve readability – e.g., ‘observational designs were included, which used sEMG in patients diagnosed with ALS, comparing them or not with people without the disease’

*Thank you for the suggestion. The abstract was revised to improve readability, and the sentence was rewritten to clearly indicate that observational studies using sEMG in individuals with ALS, with or without control groups, were included.*

2. In the intro: ‘over other neuromuscular assessment methods, such as dynamometry [11] and invasive sEMG [12]..’ sEMG should be EMG

*The term was corrected to “invasive EMG,” as recommended.*

3. In the intro: ‘physical properties of the electrodes and equipment, as well as aspects related to the calibration, acquisition, recording, amplification, normalization, and processing of electromyographic signals.’ – filtering should also be mentioned (and this would be a good place to include consideration of established guidelines)

*We incorporated “filtering” among the signal-processing steps and added references to the relevant guidelines at that point in the text.*

4. I was curious as to why the study of the respiratory muscles was excluded? Assessment of these muscles is very important, and this could contribute to the lack of studies assessing the torso that were included in the review process.

*We thank the reviewer for this question. Respiratory muscles were excluded based on eligibility criteria defined a priori. Our focus was on voluntary skeletal muscle activation during motor or functional tasks. Many respiratory studies address ventilatory physiology, ventilatory support, invasive protocols, or predominantly involuntary contractions, which fall outside the intended scope. A clear justification was added to the Methods.*

5. Results: In ‘Targeted muscles’ – ‘some studies have investigated more complex electromyographic patterns, including regions of the torso and cervical’. Do you mean some studies have studied more complex regions? If not, please explain why the sEMG patterns are more complex in the stated body regions compared to others. For example, are you considering the ECG artefact?

*Thank you for the observation. This section was rewritten to explain why these regions are considered more complex for analysis: deeper and multilayer musculature, influence of respiratory and postural mechanics, and greater susceptibility to artifacts, including ECG. The updated explanation is presented in the “Targeted Muscles” subsection on page 10, second paragraph.*

6. Results: In ‘Evaluation’ – the rest periods are described as adequate. The research question states the duration of the rest periods would be reported, so more specific detail is required here to answer the question (or highlight if duration of rest was not reported).

*We revised the “Evaluation” subsection to provide detailed information on rest-period duration when reported by the studies. When this information was not provided by the authors, this is now explicitly stated. This update makes the answer to RQ4 more complete and transparent, and the revised content appears on page 10.*

7. Results: In ‘sEMG properties’ the following should be rewritten in english ‘Grades de 64 eletrodos circulares (8×8),…’
Also - trend-free fluctuation analysis (DFA), - should be ‘detrended fluctuation analysis’
Results: RQ8, when interventions have been done does not seem to have been answered.

*The electrode-array description was fully rewritten in English, as presented on page 11 (third paragraph). The term “Trend-free fluctuation analysis (DFA)” was corrected to “detrended fluctuation analysis,” with the updated wording appearing on page 12 (first paragraph). We also incorporated the missing information related to RQ8, specifying when interventions occurred; this addition is found on page 13.*

8. Should references in the appendices be numbered so they directly link to the manuscript reference list?

*The references in the appendices were numbered to directly correspond with the main reference list.*

9. Appendix 10 – ‘Intervention results’ does not seem to be the correct heading for the table, is this not ‘study observations’?

*The heading was corrected to “Study observations,” better reflecting the content.*

10. The conclusion is slightly repetitive of the final discussion paragraph, so could be made more concise. I was also struck by the statement that the review showed wide applications of sEMG, given that 34 papers were identified from a nearly 30-year period. While it is clear the number of studies in this area is growing, this struck me as quite a small number maybe indicating that sEMG is relatively underexplored in ALS – which again would be a valuable message for the research community to appreciate.

*We revised the conclusion to remove redundancies and ensure greater conciseness. The phrase regarding the “wide applications” of sEMG was reformulated to clarify that, despite the growing use of the technique, the relatively small number of studies over nearly three decades suggests that sEMG remains underexplored in ALS, a key message highlighted in the revised text. This update appears on page 15*

Reviewer #2: The abstract includes statements “These findings showed sensitivity to the progression of the disease, which may contribute to early diagnosis, phenotypic stratification, and functional monitoring of ALS patients” and “with the potential to increase diagnostic accuracy and support new therapeutic strategies based on electrophysiological biomarkers.” However, these claims are not adequately supported or elaborated in the Results or Discussion sections. The manuscript would benefit from either providing evidence-based discussion to support these arguments, or softening the language in the abstract to better align with the presented evidence.

In addition, the review does not address intermuscular coherence (IMC)—a surface EMG–based technique that has been used to assess upper motor neuron involvement in ALS and corticospinal tract integrity.

*We thank the reviewer for this insightful and constructive comment.*

*Regarding the abstract, we agree that the original wording could imply stronger claims than those directly supported by the data presented in the Results and Discussion sections. To address this concern, we have revised the abstract to soften the language, ensuring that the statements now better reflect the exploratory nature of the findings and their potential implications, rather than definitive clinical applications. Specifically, claims related to early diagnosis, phenotypic stratification, diagnostic accuracy, and therapeutic strategies were reformulated to emphasize potential relevance and future perspectives, aligned with the evidence discussed in the manuscript.*

*Concerning intermuscular coherence (IMC), we acknowledge that the review did not explicitly use this terminology in its initial version. We would like to clarify that the study by Quintão et al., 2021—which indeed presents an analysis of intermuscular coherence—was included in our review. However, it was originally referred to under the broader term “sEMG behavior.” To improve conceptual clarity and precision, we have revised the manuscript to explicitly refer to this analysis as “intermuscular coherence (IMC)”, ensuring that the terminology accurately reflects the methodological approach and aligns with current literature.*
