# Supplementary material for: Applications of electromyography in Amyotrophic Lateral Sclerosis: A systematic review
Source: PLoS One. 2026 Jun 22;21(6):e0350029. doi: 10.1371/journal.pone.0350029 (PMC13286138; doi:10.1371/journal.pone.0350029)
Supplement: Peer review history

# Peer review: Decision Letter 0

**DOI:** 10.1371/journal.pone.0350029.r001

**Authors:** Simone Ranaldi

5 Nov 2025

PONE-D-25-34529Electromyography as a Tool to Motion Analysis for People with Amyotrophic Lateral Sclerosis: a systematic reviewPLOS ONE

Dear Dr. Rodrigues Lindquist,

Thank you for submitting your manuscript to PLOS ONE. After careful consideration, we feel that it has merit but does not fully meet PLOS ONE’s publication criteria as it currently stands. Therefore, we invite you to submit a revised version of the manuscript that addresses the points raised during the review process.

Please submit your revised manuscript by Dec 20 2025 11:59PM. If you will need more time than this to complete your revisions, please reply to this message or contact the journal office at plosone@plos.org. When you're ready to submit your revision, log on to https://www.editorialmanager.com/pone/ and select the 'Submissions Needing Revision' folder to locate your manuscript file.

Please include the following items when submitting your revised manuscript:

- A rebuttal letter that responds to each point raised by the academic editor and reviewer(s). You should upload this letter as a separate file labeled 'Response to Reviewers'.
- A marked-up copy of your manuscript that highlights changes made to the original version. You should upload this as a separate file labeled 'Revised Manuscript with Track Changes'.
- An unmarked version of your revised paper without tracked changes. You should upload this as a separate file labeled 'Manuscript'.

If you would like to make changes to your financial disclosure, please include your updated statement in your cover letter. Guidelines for resubmitting your figure files are available below the reviewer comments at the end of this letter.

If applicable, we recommend that you deposit your laboratory protocols in protocols.io to enhance the reproducibility of your results. Protocols.io assigns your protocol its own identifier (DOI) so that it can be cited independently in the future. For instructions see: https://journals.plos.org/plosone/s/submission-guidelines#loc-laboratory-protocols. Additionally, PLOS ONE offers an option for publishing peer-reviewed Lab Protocol articles, which describe protocols hosted on protocols.io. Read more information on sharing protocols at https://plos.org/protocols?utm_medium=editorial-email&utm_source=authorletters&utm_campaign=protocols.

We look forward to receiving your revised manuscript.

Kind regards,

Simone Ranaldi, Ph.D.

Academic Editor

PLOS ONE

Journal Requirements:

When submitting your revision, we need you to address these additional requirements.

1. Please ensure that your manuscript meets PLOS ONE's style requirements, including those for file naming. The PLOS ONE style templates can be found at https://journals.plos.org/plosone/s/file?id=wjVg/PLOSOne_formatting_sample_main_body.pdf and https://journals.plos.org/plosone/s/file?id=ba62/PLOSOne_formatting_sample_title_authors_affiliations.pdf

2. When completing the data availability statement of the submission form, you indicated that you will make your data available on acceptance. We strongly recommend all authors decide on a data sharing plan before acceptance, as the process can be lengthy and hold up publication timelines. Please note that, though access restrictions are acceptable now, your entire data will need to be made freely accessible if your manuscript is accepted for publication. This policy applies to all data except where public deposition would breach compliance with the protocol approved by your research ethics board. If you are unable to adhere to our open data policy, please kindly revise your statement to explain your reasoning and we will seek the editor's input on an exemption. Please be assured that, once you have provided your new statement, the assessment of your exemption will not hold up the peer review process.

3. We note that Figure 3 in your submission contain [map/satellite] images which may be copyrighted. All PLOS content is published under the Creative Commons Attribution License (CC BY 4.0), which means that the manuscript, images, and Supporting Information files will be freely available online, and any third party is permitted to access, download, copy, distribute, and use these materials in any way, even commercially, with proper attribution. For these reasons, we cannot publish previously copyrighted maps or satellite images created using proprietary data, such as Google software (Google Maps, Street View, and Earth). For more information, see our copyright guidelines: http://journals.plos.org/plosone/s/licenses-and-copyright.

We require you to either (1) present written permission from the copyright holder to publish these figures specifically under the CC BY 4.0 license, or (2) remove the figures from your submission:

a. You may seek permission from the original copyright holder of Figure 3 to publish the content specifically under the CC BY 4.0 license.

We recommend that you contact the original copyright holder with the Content Permission Form (http://journals.plos.org/plosone/s/file?id=7c09/content-permission-form.pdf) and the following text:

“I request permission for the open-access journal PLOS ONE to publish XXX under the Creative Commons Attribution License (CCAL) CC BY 4.0 (http://creativecommons.org/licenses/by/4.0/). Please be aware that this license allows unrestricted use and distribution, even commercially, by third parties. Please reply and provide explicit written permission to publish XXX under a CC BY license and complete the attached form.”

Please upload the completed Content Permission Form or other proof of granted permissions as an "Other" file with your submission.

In the figure caption of the copyrighted figure, please include the following text: “Reprinted from [ref] under a CC BY license, with permission from [name of publisher], original copyright [original copyright year].”

b. If you are unable to obtain permission from the original copyright holder to publish these figures under the CC BY 4.0 license or if the copyright holder’s requirements are incompatible with the CC BY 4.0 license, please either i) remove the figure or ii) supply a replacement figure that complies with the CC BY 4.0 license. Please check copyright information on all replacement figures and update the figure caption with source information. If applicable, please specify in the figure caption text when a figure is similar but not identical to the original image and is therefore for illustrative purposes only.

The following resources for replacing copyrighted map figures may be helpful:

USGS National Map Viewer (public domain): http://viewer.nationalmap.gov/viewer/

The Gateway to Astronaut Photography of Earth (public domain): http://eol.jsc.nasa.gov/sseop/clickmap/

Maps at the CIA (public domain): https://www.cia.gov/library/publications/the-world-factbook/index.html and https://www.cia.gov/library/publications/cia-maps-publications/index.html

NASA Earth Observatory (public domain): http://earthobservatory.nasa.gov/

Landsat: http://landsat.visibleearth.nasa.gov/

USGS EROS (Earth Resources Observatory and Science (EROS) Center) (public domain): http://eros.usgs.gov/#

Natural Earth (public domain): http://www.naturalearthdata.com/

4. Please include your tables as part of your main manuscript and remove the individual files. Please note that supplementary tables (should remain/ be uploaded) as separate "supporting information" files.

5. Please include captions for your Supporting Information files at the end of your manuscript, and update any in-text citations to match accordingly. Please see our Supporting Information guidelines for more information: http://journals.plos.org/plosone/s/supporting-information.

6. We are unable to open your Supporting Information file [bmc_article.bib, plos2015.bst, plos_latex_template.tex]. Please kindly revise as necessary and re-upload.

7. If the reviewer comments include a recommendation to cite specific previously published works, please review and evaluate these publications to determine whether they are relevant and should be cited. There is no requirement to cite these works unless the editor has indicated otherwise.

Additional Editor Comments :

The reports from two independent reviewers identified some major issues in the structure and the discussion of the material under analysis. For this reason, the manuscript needs a major revision before it can be considered for publication.

[Note: HTML markup is below. Please do not edit.]

Reviewers' comments:

Reviewer's Responses to Questions

**Comments to the Author**

1. Is the manuscript technically sound, and do the data support the conclusions?

The manuscript must describe a technically sound piece of scientific research with data that supports the conclusions. Experiments must have been conducted rigorously, with appropriate controls, replication, and sample sizes. The conclusions must be drawn appropriately based on the data presented.

Reviewer #1: Partly

Reviewer #2: Partly

**********

2. Has the statistical analysis been performed appropriately and rigorously?

Reviewer #1: N/A

Reviewer #2: Yes

**********

3. Have the authors made all data underlying the findings in their manuscript fully available?

The PLOS Data policy requires authors to make all data underlying the findings described in their manuscript fully available without restriction, with rare exception (please refer to the Data Availability Statement in the manuscript PDF file). The data should be provided as part of the manuscript or its supporting information, or deposited to a public repository. For example, in addition to summary statistics, the data points behind means, medians and variance measures should be available. If there are restrictions on publicly sharing data—e.g. participant privacy or use of data from a third party—those must be specified.

Reviewer #1: Yes

Reviewer #2: Yes

**********

4. Is the manuscript presented in an intelligible fashion and written in standard English?

PLOS ONE does not copyedit accepted manuscripts, so the language in submitted articles must be clear, correct, and unambiguous. Any typographical or grammatical errors should be corrected at revision, so please note any specific errors here.

Reviewer #1: Yes

Reviewer #2: Yes

**********

5. Review Comments to the Author

Please use the space provided to explain your answers to the questions above. You may also include additional comments for the author, including concerns about dual publication, research ethics, or publication ethics. (Please upload your review as an attachment if it exceeds 20,000 characters)

Reviewer #1: The manuscript describes a systematic review of the application of surface EMG for the assessment of neuromuscular features in people diagnosed with ALS. Overall it is clear a significant amount of work has been completed, and a summary of the approaches and potential application of sEMG in this clinical population is provided. It is good to see the review protocol was previously published and made available.

From the evidence in the manuscript, it seems the work is of reasonably high quality. However, the work has not considered current guidelines for use of sEMG in its assessment approach. This seems like a very large omission which reduces the value of the work presented and also means the review does not evaluate the literature within any established framework of currently accepted standards.

In addition, there are other areas of the manuscript which could be improved to make the information gathered more accessible to readers. In particular, there are 10 appendices that the reader must download to see the full details of information summarised in the manuscript. This feels quite excessive, and I wonder how many people will access these and fully appreciate (or use) the breadth of information that has been gathered and presented.

The main points I recommend be addressed are:

1. The title does not accurately reflect the topic of the review and should be amended. Specifically, motion analysis is not a part of the review and should therefore not be mentioned.

2. The introduction refers to difficulty in establishing standardised guidelines, but there is no mention in the manuscript about guidelines for sEMG use that have already been established. These should, at least be mentioned, and in the best case I think it would be valuable if the reported studies were assessed by these guidelines. Guidelines to consider are: SENIAM (http://seniam.org/, https://pubmed.ncbi.nlm.nih.gov/11018445/ ) and CEDE (https://www.sciencedirect.com/special-issue/10CFPDX6CN9 ). The lack of consideration of these standards reduces the value of the review, and misses an opportunity to highlight the availability of these standards to the ALS research and clinical community.

3. The research questions would benefit from some refinement. Particularly, the term clinical is used a lot, but this does not always seem appropriate. For example, RQ2 asks what the clinical characteristics of the participants are reported. The information includes age, which is not specifically a clinical feature. Inter-related with this point (and see note on risk of bias below), other features such as sex characteristics are not included. What is the rationale for not including this?

Additionally, RQ5 asks about the ‘technical properties of sEMG’ – it seems it would be more appropriate that this stated ‘what hardware has been used for sEMG acquisition’ – or similar. RQ8 asks whether ‘biofeedback’ was used during ‘interventions’ and here interventions seems to reflect the completion of the study or experiment within an observational study design (i.e., the neuromuscular properties are being observed). I appreciate this second point is maybe more minor, but I think it is important in terms of ensuring correct and appropriate language is used throughout to improve clarity of the manuscript.

4. It would be helpful to the reader to at least have a summary of the search strategy in this manuscript. Otherwise you are demanding people download an additional article to understand the work that has been conducted. I think it is better if this manuscript can standalone and be a self-contained piece of work.

5. The methodological quality assessment is described as part of the data analysis process in the methods, but in the results section it seems to be stated that this was part of the selection process ‘Following eligibility assessment and methodological quality evaluation, 34 studies were included in the quantitative synthesis.’ It should be made clear in the methods how this assessment was completed, and if it was solely for study selection purposes the threshold or criterion for inclusion/exclusion should be explained.

6. The methods describe a risk of bias assessment as having been completed as part of data analysis, but the results of this assessment are not reported. The prisma checklist also states this was not done. As the aim of the review seems to be to highlight the value of sEMG in ALS neuromuscular assessment this seems important to have completed and be reported.

7. It seemed to that much of the information in appendices could have been included in the main manuscript so the reader has fuller insight into the studies assessed. For example, could details in appendices 3, 4, and 8 not be presented as pie charts? Could the table in appendix 9 not be in the main text? Are there publisher guidelines which prevent more tables/figures being included?

8. There are inconsistencies in the details presented across some of the tables in the appendices.

For example, appendix 6 – SpiQE is reported as being both an analysis technique and an algorithm for some entries and just as an analysis technique in others (with algorithm stated as ‘not mentioned’). This also led to me asking how have you defined ‘analysis technique’ and ‘algorithm’ – what is the distinction between them? In the manuscript text the software used is not reported, but it would be useful to have a sense of what has been used without having to download the appendix. Equally, it would also be valuable to know which of the algorithms are commercially available and which available open source.

Further example, appendix 5 – REFA is defined as both a brand and an amplifier. Where it is entered as a brand the statement under ‘amplifier’ is ‘not mentioned’.

In appendix 7 – why is the distinction between ‘other devices’ and ‘neuroimaging’ important? Is neuroimaging not simply another device? I ask this because stating ‘Not mentioned’ could be seen as a negative for the study, but in this case it reflects the fact that neuroimaging was not appropriate to address that study’s specific research question.

More minor points:

1. Text in the abstract could be rephrased to improve readability – e.g., ‘observational designs were included, which used sEMG in patients diagnosed with ALS, comparing them or not with people without the disease’

2. In the intro: ‘over other neuromuscular assessment methods, such as dynamometry [11] and invasive sEMG [12]..’ sEMG should be EMG

3. In the intro: ‘physical properties of the electrodes and equipment, as well as aspects related to the calibration, acquisition, recording, amplification, normalization, and processing of electromyographic signals.’ – filtering should also be mentioned (and this would be a good place to include consideration of established guidelines)

4. I was curious as to why the study of the respiratory muscles was excluded? Assessment of these muscles is very important, and this could contribute to the lack of studies assessing the torso that were included in the review process.

5. Results: In ‘Targeted muscles’ – ‘some studies have investigated more complex electromyographic patterns, including regions of the torso and cervical’. Do you mean some studies have studied more complex regions? If not, please explain why the sEMG patterns are more complex in the stated body regions compared to others. For example, are you considering the ECG artefact?

6. Results: In ‘Evaluation’ – the rest periods are described as adequate. The research question states the duration of the rest periods would be reported, so more specific detail is required here to answer the question (or highlight if duration of rest was not reported).

7. Results: In ‘sEMG properties’ the following should be rewritten in english ‘Grades de 64 eletrodos circulares (8×8),…’

Also - trend-free fluctuation analysis (DFA), - should be ‘detrended fluctuation analysis’

Results: RQ8, when interventions have been done does not seem to have been answered.

8. Should references in the appendices be numbered so they directly link to the manuscript reference list?

9. Appendix 10 – ‘Intervention results’ does not seem to be the correct heading for the table, is this not ‘study observations’?

10. The conclusion is slightly repetitive of the final discussion paragraph, so could be made more concise. I was also struck by the statement that the review showed wide applications of sEMG, given that 34 papers were identified from a nearly 30-year period. While it is clear the number of studies in this area is growing, this struck me as quite a small number maybe indicating that sEMG is relatively underexplored in ALS – which again would be a valuable message for the research community to appreciate.

Reviewer #2: The abstract includes statements “These findings showed sensitivity to the progression of the disease, which may contribute to early diagnosis, phenotypic stratification, and functional monitoring of ALS patients” and “with the potential to increase diagnostic accuracy and support new therapeutic strategies based on electrophysiological biomarkers.” However, these claims are not adequately supported or elaborated in the Results or Discussion sections. The manuscript would benefit from either providing evidence-based discussion to support these arguments, or softening the language in the abstract to better align with the presented evidence.

In addition, the review does not address intermuscular coherence (IMC)—a surface EMG–based technique that has been used to assess upper motor neuron involvement in ALS and corticospinal tract integrity.

Minor:

**********

6. PLOS authors have the option to publish the peer review history of their article (what does this mean?). If published, this will include your full peer review and any attached files.

If you choose “no”, your identity will remain anonymous but your review may still be made public.

**Do you want your identity to be public for this peer review?**   For information about this choice, including consent withdrawal, please see our Privacy Policy.

Reviewer #1: No

Reviewer #2: No

**********

[NOTE: If reviewer comments were submitted as an attachment file, they will be attached to this email and accessible via the submission site. Please log into your account, locate the manuscript record, and check for the action link "View Attachments". If this link does not appear, there are no attachment files.]

To ensure your figures meet our technical requirements, please review our figure guidelines: https://journals.plos.org/plosone/s/figures

You may also use PLOS’s free figure tool, NAAS, to help you prepare publication quality figures: https://journals.plos.org/plosone/s/figures#loc-tools-for-figure-preparation.

NAAS will assess whether your figures meet our technical requirements by comparing each figure against our figure specifications.

# Author comment: Author response to Decision Letter 1

**DOI:** 10.1371/journal.pone.0350029.r002

7 Jan 2026

Dear Reviewers,

We sincerely thank you for the time, attention, and thoughtful feedback provided during the evaluation of our manuscript. Your detailed comments greatly contributed to strengthening the clarity, rigor, and methodological consistency of this systematic review. Below, we present a point-by-point response to each comment, describing the revisions implemented and clarifying the decisions made during the review process.

1. The title does not accurately reflect the topic of the review and should be amended. Specifically, motion analysis is not a part of the review and should therefore not be mentioned.

We appreciate the observation. The title has been revised as suggested to more accurately reflect the scope of the review.

Revised title: “Applications of Electromyography in Amyotrophic Lateral Sclerosis: A Systematic Review.”

2. The introduction refers to difficulty in establishing standardised guidelines, but there is no mention in the manuscript about guidelines for sEMG use that have already been established. These should, at least be mentioned, and in the best case I think it would be valuable if the reported studies were assessed by these guidelines. Guidelines to consider are: SENIAM (http://seniam.org/, https://pubmed.ncbi.nlm.nih.gov/11018445/ ) and CEDE (https://www.sciencedirect.com/special-issue/10CFPDX6CN9 ). The lack of consideration of these standards reduces the value of the review, and misses an opportunity to highlight the availability of these standards to the ALS research and clinical community.

We thank the reviewer for this observation. We revised the Introduction and added a dedicated paragraph on page 2, paragraph 6, explicitly addressing the SENIAM and CEDE guidelines and emphasizing their relevance for the standardization of electrode placement, acquisition parameters, and signal-processing procedures. We also discuss how the lack of adherence to these guidelines contributes to methodological heterogeneity and constitutes an important limitation among the included studies. This update strengthens the methodological context of the review and fully addresses the reviewer’s recommendation.

3. The research questions would benefit from some refinement. Particularly, the term clinical is used a lot, but this does not always seem appropriate. For example, RQ2 asks what the clinical characteristics of the participants are reported. The information includes age, which is not specifically a clinical feature. Inter-related with this point (and see note on risk of bias below), other features such as sex characteristics are not included. What is the rationale for not including this?

Additionally, RQ5 asks about the ‘technical properties of sEMG’ – it seems it would be more appropriate that this stated ‘what hardware has been used for sEMG acquisition’ – or similar. RQ8 asks whether ‘biofeedback’ was used during ‘interventions’ and here interventions seems to reflect the completion of the study or experiment within an observational study design (i.e., the neuromuscular properties are being observed). I appreciate this second point is maybe more minor, but I think it is important in terms of ensuring correct and appropriate language is used throughout to improve clarity of the manuscript.

The research questions were revised as suggested to improve terminological precision and clarity. We removed the term “clinical” where it was not appropriate, simplified the question regarding participant characteristics, reformulated RQ5 to explicitly address hardware and acquisition parameters, and adjusted RQ8 to avoid the incorrect use of the term “interventions” in observational study designs. We also refined the wording of the remaining questions to ensure greater objectivity and consistency with recommended terminology. All modifications are reflected in the updated Research Questions Table, now presented on page 4.

4. It would be helpful to the reader to at least have a summary of the search strategy in this manuscript. Otherwise you are demanding people download an additional article to understand the work that has been conducted. I think it is better if this manuscript can standalone and be a self-contained piece of work.

We thank the reviewer for this important suggestion. In response, we incorporated a concise summary of the search strategy directly into the Methods section, ensuring that the manuscript is self-contained and does not require consultation of external documents. These additions, which include the keywords and Boolean operators, are now presented on page 5. This revision improves the transparency and completeness of the manuscript.

5. The assessment of methodological quality is described as part of the data analysis process in the methods section, but in the results section it appears to be stated that this was part of the selection process: “After eligibility assessment and methodological quality assessment, 34 studies were included in the quantitative analysis.” It should be clear in the methods section how this assessment was carried out and, if it was solely for the purpose of study selection, the inclusion/exclusion limits or thresholds should be explained.

We thank the reviewer for this observation. The Methods section has been revised to clarify that both the methodological quality assessment (GRADE) and the risk of bias assessment (PROBAST) were used as eligibility criteria. We now explicitly state that studies classified as having very low evidence by GRADE or that present a high risk of bias in any domain of PROBAST were excluded from the final synthesis. These clarifications have been added on page 7, in the Data Analysis section.

We are presenting below the image referring to the PROBAST of the included articles.

6. The methods describe a risk of bias assessment as having been completed as part of data analysis, but the results of this assessment are not reported. The prisma checklist also states this was not done. As the aim of the review seems to be to highlight the value of sEMG in ALS neuromuscular assessment this seems important to have completed and be reported.

We thank the reviewer for this important observation. We clarified in the Methods section that the risk of bias assessment using PROBAST was employed as an eligibility criterion, rather than as a post-selection analytical step. Only studies classified as having low risk of bias across all PROBAST domains were included in the final synthesis; therefore, no studies with high or unclear risk of bias were retained, which explains why no risk-of-bias results appeared in the original Results section. This information is now more clearly presented on page 7, in the Data Analysis section.

7. It seemed to that much of the information in appendices could have been included in the main manuscript so the reader has fuller insight into the studies assessed. For example, could details in appendices 3, 4, and 8 not be presented as pie charts? Could the table in appendix 9 not be in the main text? Are there publisher guidelines which prevent more tables/figures being included?

We thank the reviewer for this suggestion. In response, we have added figures to the main manuscript summarizing the muscles analyzed and the types of feedback/outcomes assessed, which condense information previously presented in the appendices and improve clarity for the reader. The remaining appendices were maintained to preserve detailed study-level information without overloading the main text.

8. There are inconsistencies in the details presented across some of the tables in the appendices.

For example, appendix 6 – SpiQE is reported as being both an analysis technique and an algorithm for some entries and just as an analysis technique in others (with algorithm stated as ‘not mentioned’). This also led to me asking how have you defined ‘analysis technique’ and ‘algorithm’ – what is the distinction between them? In the manuscript text the software used is not reported, but it would be useful to have a sense of what has been used without having to download the appendix. Equally, it would also be valuable to know which of the algorithms are commercially available and which available open source.

Further example, appendix 5 – REFA is defined as both a brand and an amplifier. Where it is entered as a brand the statement under ‘amplifier’ is ‘not mentioned’.

In appendix 7 – why is the distinction between ‘other devices’ and ‘neuroimaging’ important? Is neuroimaging not simply another device? I ask this because stating ‘Not mentioned’ could be seen as a negative for the study, but in this case it reflects the fact that neuroimaging was not appropriate to address that study’s specific research question.

We have revised all tables and appendices for inconsistencies. In regard to Appendix 6, our initial idea in having two columns, one for analysis technique and one for algorithms, was to provide more information about the processing pipeline without having too much information on a single column. However, we agree that the distinction between the two terms is not clear in the manuscript, and thus decided to join both columns into a single one: analysis technique. We have integrated the infformation from both columns accordingly.

In Appendix 5, REFA is the name of the device, while TMSi is the brand of the device. TMS International BV was rebranded as "TMSi — an Artinis company"; however, we chose to maintain the legacy name in the corresponding entries as they are reported as so in the collected manuscripts. We have corrected all inconsistencies related to these entries.

As for Appendix 7, the rationale behind this division was similar to that employed in Appendix 6, i.e., to have more granularity in the described data. As such, we have also joined both columns into a single one without loss of information.

More minor points:

1. Text in the abstract could be rephrased to improve readability – e.g., ‘observational designs were included, which used sEMG in patients diagnosed with ALS, comparing them or not with people without the disease’

Thank you for the suggestion. The abstract was revised to improve readability, and the sentence was rewritten to clearly indicate that observational studies using sEMG in individuals with ALS, with or without control groups, were included.

2. In the intro: ‘over other neuromuscular assessment methods, such as dynamometry [11] and invasive sEMG [12]..’ sEMG should be EMG

The term was corrected to “invasive EMG,” as recommended.

3. In the intro: ‘physical properties of the electrodes and equipment, as well as aspects related to the calibration, acquisition, recording, amplification, normalization, and processing of electromyographic signals.’ – filtering should also be mentioned (and this would be a good place to include consideration of established guidelines)

We incorporated “filtering” among the signal-processing steps and added references to the relevant guidelines at that point in the text.

4. I was curious as to why the study of the respiratory muscles was excluded? Assessment of these muscles is very important, and this could contribute to the lack of studies assessing the torso that were included in the review process.

We thank the reviewer for this question. Respiratory muscles were excluded based on eligibility criteria defined a priori. Our focus was on voluntary skeletal muscle activation during motor or functional tasks. Many respiratory studies address ventilatory physiology, ventilatory support, invasive protocols, or predominantly involuntary contractions, which fall outside the intended scope. A clear justification was added to the Methods.

5. Results: In ‘Targeted muscles’ – ‘some studies have investigated more complex electromyographic patterns, including regions of the torso and cervical’. Do you mean some studies have studied more complex regions? If not, please explain why the sEMG patterns are more complex in the stated body regions compared to others. For example, are you considering the ECG artefact?

Thank you for the observation. This section was rewritten to explain why these regions are considered more complex for analysis: deeper and multilayer musculature, influence of respiratory and postural mechanics, and greater susceptibility to artifacts, including ECG. The updated explanation is presented in the “Targeted Muscles” subsection on page 10, second paragraph.

6. Results: In ‘Evaluation’ – the rest periods are described as adequate. The research question states the duration of the rest periods would be reported, so more specific detail is required here to answer the question (or highlight if duration of rest was not reported).

We revised the “Evaluation” subsection to provide detailed information on rest-period duration when reported by the studies. When this information was not provided by the authors, this is now explicitly stated. This update makes the answer to RQ4 more complete and transparent, and the revised content appears on page 10.

7. Results: In ‘sEMG properties’ the following should be rewritten in english ‘Grades de 64 eletrodos circulares (8×8),…’

Also - trend-free fluctuation analysis (DFA), - should be ‘detrended fluctuation analysis’

Results: RQ8, when interventions have been done does not seem to have been answered.

The electrode-array description was fully rewritten in English, as presented on page 11 (third paragraph). The term “Trend-free fluctuation analysis (DFA)” was corrected to “detrended fluctuation analysis,” with the updated wording appearing on page 12 (first paragraph). We also incorporated the missing information related to RQ8, specifying when interventions occurred; this addition is found on page 13.

8. Should references in the appendices be numbered so they directly link to the manuscript reference list?

The references in the appendices were numbered to directly correspond with the main reference list.

9. Appendix 10 – ‘Intervention results’ does not seem to be the correct heading for the table, is this not ‘study observations’?

The heading was corrected to “Study observations,” better reflecting the content.

10. The conclusion is slightly repetitive of the final discussion paragraph, so could be made more concise. I was also struck by the statement that the review showed wide applications of sEMG, given that 34 papers were identified from a nearly 30-year period. While it is clear the number of studies in this area is growing, this struck me as quite a small number maybe indicating that sEMG is relatively underexplored in ALS – which again would be a valuable message for the research community to appreciate.

We revised the conclusion to remove redundancies and ensure greater conciseness. The phrase regarding the “wide applications” of sEMG was reformulated to clarify that, despite the growing use of the technique, the relatively small number of studies over nearly three decades suggests that sEMG remains underexplored in ALS, a key message highlighted in the revised text. This update appears on page 15

Reviewer #2: The abstract includes statements “These findings showed sensitivity to the progression of the disease, which may contribute to early diagnosis, phenotypic stratification, and functional monitoring of ALS patients” and “with the potential to increase diagnostic accuracy and support new therapeutic strategies based on electrophysiological biomarkers.” However, these claims are not adequately supported or elaborated in the Results or Discussion sections. The manuscript would benefit from either providing evidence-based discussion to support these arguments, or softening the language in the abstract to better align with the presented evidence.

In addition, the review does not address intermuscular coherence (IMC)—a surface EMG–based technique that has been used to assess upper motor neuron involvement in ALS and corticospinal tract integrity.

We thank the reviewer for this insightful and constructive comment.

Regarding the abstract, we agree that the original wording could imply stronger claims than those directly supported by the data presented in the Results and Discussion sections. To address this concern, we have revised the abstract to soften the language, ensuring that the statements now better reflect

Attachment Submitted filename: Response Letter.docx

# Peer review: Decision Letter 1

**DOI:** 10.1371/journal.pone.0350029.r003

**Authors:** Simone Ranaldi, Simone Ranaldi

11 Mar 2026

PONE-D-25-34529R1Applications of Electromyography in Amyotrophic Lateral Sclerosis: A Systematic ReviewPLOS One

Dear Dr. Rodrigues Lindquist,

Thank you for submitting your manuscript to PLOS ONE. After careful consideration, we feel that it has merit but does not fully meet PLOS ONE’s publication criteria as it currently stands. Therefore, we invite you to submit a revised version of the manuscript that addresses the points raised during the review process.

Please submit your revised manuscript by Apr 25 2026 11:59PM. If you will need more time than this to complete your revisions, please reply to this message or contact the journal office at plosone@plos.org. When you're ready to submit your revision, log on to https://www.editorialmanager.com/pone/ and select the 'Submissions Needing Revision' folder to locate your manuscript file.

Please include the following items when submitting your revised manuscript:

- A letter that responds to each point raised by the academic editor and reviewer(s). You should upload this letter as a separate file labeled 'Response to Reviewers'.
- A marked-up copy of your manuscript that highlights changes made to the original version. You should upload this as a separate file labeled 'Revised Manuscript with Track Changes'.
- An unmarked version of your revised paper without tracked changes. You should upload this as a separate file labeled 'Manuscript'.

If you would like to make changes to your financial disclosure, please include your updated statement in your cover letter. Guidelines for resubmitting your figure files are available below the reviewer comments at the end of this letter.

If applicable, we recommend that you deposit your laboratory protocols in protocols.io to enhance the reproducibility of your results. Protocols.io assigns your protocol its own identifier (DOI) so that it can be cited independently in the future. For instructions see: https://journals.plos.org/plosone/s/submission-guidelines#loc-laboratory-protocols. Additionally, PLOS ONE offers an option for publishing peer-reviewed Lab Protocol articles, which describe protocols hosted on protocols.io. Read more information on sharing protocols at https://plos.org/protocols?utm_medium=editorial-email&utm_source=authorletters&utm_campaign=protocols.

We look forward to receiving your revised manuscript.

Kind regards,

Simone Ranaldi, Ph.D.

Academic Editor

PLOS One

Journal Requirements:

1. If the reviewer comments include a recommendation to cite specific previously published works, please review and evaluate these publications to determine whether they are relevant and should be cited. There is no requirement to cite these works unless the editor has indicated otherwise.

2. Please review your reference list to ensure that it is complete and correct. If you have cited papers that have been retracted, please include the rationale for doing so in the manuscript text, or remove these references and replace them with relevant current references. Any changes to the reference list should be mentioned in the rebuttal letter that accompanies your revised manuscript. If you need to cite a retracted article, indicate the article’s retracted status in the References list and also include a citation and full reference for the retraction notice.

[Note: HTML markup is below. Please do not edit.]

Reviewers' comments:

Reviewer's Responses to Questions

**Comments to the Author**

1. If the authors have adequately addressed your comments raised in a previous round of review and you feel that this manuscript is now acceptable for publication, you may indicate that here to bypass the “Comments to the Author” section, enter your conflict of interest statement in the “Confidential to Editor” section, and submit your "Accept" recommendation.

Reviewer #1: (No Response)

Reviewer #2: All comments have been addressed

**********

2. Is the manuscript technically sound, and do the data support the conclusions?

The manuscript must describe a technically sound piece of scientific research with data that supports the conclusions. Experiments must have been conducted rigorously, with appropriate controls, replication, and sample sizes. The conclusions must be drawn appropriately based on the data presented.

Reviewer #1: Yes

Reviewer #2: Yes

**********

3. Has the statistical analysis been performed appropriately and rigorously?

Reviewer #1: N/A

Reviewer #2: Yes

**********

4. Have the authors made all data underlying the findings in their manuscript fully available?

The PLOS Data policy requires authors to make all data underlying the findings described in their manuscript fully available without restriction, with rare exception (please refer to the Data Availability Statement in the manuscript PDF file). The data should be provided as part of the manuscript or its supporting information, or deposited to a public repository. For example, in addition to summary statistics, the data points behind means, medians and variance measures should be available. If there are restrictions on publicly sharing data—e.g. participant privacy or use of data from a third party—those must be specified.

Reviewer #1: Yes

Reviewer #2: Yes

**********

5. Is the manuscript presented in an intelligible fashion and written in standard English?

PLOS ONE does not copyedit accepted manuscripts, so the language in submitted articles must be clear, correct, and unambiguous. Any typographical or grammatical errors should be corrected at revision, so please note any specific errors here.

Reviewer #1: Yes

Reviewer #2: Yes

**********

6. Review Comments to the Author

Please use the space provided to explain your answers to the questions above. You may also include additional comments for the author, including concerns about dual publication, research ethics, or publication ethics. (Please upload your review as an attachment if it exceeds 20,000 characters)

Reviewer #1: Thank you for the detailed responses and I appreciate the way in which my initial recommendations have been incorporated into the manuscript.

I just have one very minor point that I think would further improve the manuscript, which is to ensure how well the included studies meet CEDE guidelines be discussed. Highlighting these existing standards in the introduction is good to see, but in the context of the aims of this work it is important to at least comment on how well the included papers seem to meet these standards. I appreciate this is not part of the original methodology, so a high-level discussion would be fine - for example do any of the studies mention following guidelines (publication dates allowing)?

Including this would close the loop of information and also better support statements made in the conclusion with regards the needs for standardised protocols to be adopted.

Reviewer #2: The authors have appropriately revised the manuscript and adequately addressed all of my previous comments. I have no further substantive concerns regarding the content or methodology of this review.

**********

7. PLOS authors have the option to publish the peer review history of their article (what does this mean?). If published, this will include your full peer review and any attached files.

If you choose “no”, your identity will remain anonymous but your review may still be made public.

**Do you want your identity to be public for this peer review?**   For information about this choice, including consent withdrawal, please see our Privacy Policy.

Reviewer #1: **Yes:**  Emma Hodson-Tole

Reviewer #2: **Yes:**  Kourosh Rezania

**********

[NOTE: If reviewer comments were submitted as an attachment file, they will be attached to this email and accessible via the submission site. Please log into your account, locate the manuscript record, and check for the action link "View Attachments". If this link does not appear, there are no attachment files.]

To ensure your figures meet our technical requirements, please review our figure guidelines: https://journals.plos.org/plosone/s/figures

You may also use PLOS’s free figure tool, NAAS, to help you prepare publication quality figures: https://journals.plos.org/plosone/s/figures#loc-tools-for-figure-preparation.

NAAS will assess whether your figures meet our technical requirements by comparing each figure against our figure specifications.

# Author comment: Author response to Decision Letter 2

**DOI:** 10.1371/journal.pone.0350029.r004

18 Mar 2026

Dear Editor and Reviewers,

We would like to sincerely thank the reviewers for their careful reading of our manuscript and for their constructive comments and suggestion.

Thank you for this valuable suggestion. In response, we added a brief discussion addressing how the included studies relate to established sEMG guidelines, particularly the SENIAM and CEDE recommendations.

Although the assessment of compliance with these guidelines was not part of the predefined methodological criteria of this review, we performed a qualitative appraisal of the included studies. Most articles described electrode placement following SENIAM recommendations or other standardized anatomical references. However, other methodological aspects, such as acquisition protocols, task execution, rest periods, and signal-processing procedures, varied considerably across studies, despite being clearly reported by the authors.

This observation has now been incorporated into the Discussion section to highlight that, while electrode placement often follows recognized guidelines, substantial variability remains in other stages of sEMG acquisition and analysis. This reinforces the need for broader adoption of comprehensive standardization frameworks, such as CEDE, in future ALS research. The new discussion appears on page 16, paragraph 4.

Attachment Submitted filename: Response Letter.pdf

# Peer review: Decision Letter 2

**DOI:** 10.1371/journal.pone.0350029.r005

**Authors:** Simone Ranaldi, Simone Ranaldi, Simone Ranaldi

7 May 2026

Applications of Electromyography in Amyotrophic Lateral Sclerosis: A Systematic Review

PONE-D-25-34529R2

Dear Dr. Rodrigues Lindquist,

We’re pleased to inform you that your manuscript has been judged scientifically suitable for publication and will be formally accepted for publication once it meets all outstanding technical requirements.

Within one week, you’ll receive an e-mail detailing the required amendments. When these have been addressed, you’ll receive a formal acceptance letter and your manuscript will be scheduled for publication.

An invoice will be generated when your article is formally accepted. Please note, if your institution has a publishing partnership with PLOS and your article meets the relevant criteria, all or part of your publication costs will be covered. Please make sure your user information is up-to-date by logging into Editorial Manager at Editorial Manager® and clicking the ‘Update My Information' link at the top of the page. For questions related to billing, please contact billing support.

If your institution or institutions have a press office, please notify them about your upcoming paper to help maximize its impact. If they’ll be preparing press materials, please inform our press team as soon as possible -- no later than 48 hours after receiving the formal acceptance. Your manuscript will remain under strict press embargo until 2 pm Eastern Time on the date of publication. For more information, please contact onepress@plos.org.

Kind regards,

Simone Ranaldi, Ph.D.

Academic Editor

PLOS One

Additional Editor Comments (optional):

Reviewers' comments:

Reviewer's Responses to Questions

**Comments to the Author**

1. If the authors have adequately addressed your comments raised in a previous round of review and you feel that this manuscript is now acceptable for publication, you may indicate that here to bypass the “Comments to the Author” section, enter your conflict of interest statement in the “Confidential to Editor” section, and submit your "Accept" recommendation.

Reviewer #1: All comments have been addressed

**********

2. Is the manuscript technically sound, and do the data support the conclusions?

The manuscript must describe a technically sound piece of scientific research with data that supports the conclusions. Experiments must have been conducted rigorously, with appropriate controls, replication, and sample sizes. The conclusions must be drawn appropriately based on the data presented.

Reviewer #1: Yes

**********

3. Has the statistical analysis been performed appropriately and rigorously?

Reviewer #1: N/A

**********

4. Have the authors made all data underlying the findings in their manuscript fully available?

The PLOS Data policy requires authors to make all data underlying the findings described in their manuscript fully available without restriction, with rare exception (please refer to the Data Availability Statement in the manuscript PDF file). The data should be provided as part of the manuscript or its supporting information, or deposited to a public repository. For example, in addition to summary statistics, the data points behind means, medians and variance measures should be available. If there are restrictions on publicly sharing data—e.g. participant privacy or use of data from a third party—those must be specified.

Reviewer #1: Yes

**********

5. Is the manuscript presented in an intelligible fashion and written in standard English?

PLOS ONE does not copyedit accepted manuscripts, so the language in submitted articles must be clear, correct, and unambiguous. Any typographical or grammatical errors should be corrected at revision, so please note any specific errors here.

Reviewer #1: Yes

**********

6. Review Comments to the Author

Please use the space provided to explain your answers to the questions above. You may also include additional comments for the author, including concerns about dual publication, research ethics, or publication ethics. (Please upload your review as an attachment if it exceeds 20,000 characters)

Reviewer #1: Thank you for considering my feedback and amending the manuscript. I fell this is a really nice and valuable piece of work, so congratulations on putting this together.

**********

7. PLOS authors have the option to publish the peer review history of their article (what does this mean?). If published, this will include your full peer review and any attached files.

If you choose “no”, your identity will remain anonymous but your review may still be made public.

**Do you want your identity to be public for this peer review?**   For information about this choice, including consent withdrawal, please see our Privacy Policy.

Reviewer #1: **Yes:**  Dr. Emma Hodson-Tole

**********

# Editor report: Acceptance letter

**DOI:** 10.1371/journal.pone.0350029.r006

**Authors:** Simone Ranaldi, Simone Ranaldi, Simone Ranaldi

PONE-D-25-34529R2

PLOS One

Dear Dr. Rodrigues Lindquist,

I'm pleased to inform you that your manuscript has been deemed suitable for publication in PLOS One. Congratulations! Your manuscript is now being handed over to our production team.

At this stage, our production department will prepare your paper for publication. This includes ensuring the following:

* All references, tables, and figures are properly cited

* All relevant supporting information is included in the manuscript submission,

* There are no issues that prevent the paper from being properly typeset

You will receive further instructions from the production team, including instructions on how to review your proof when it is ready. Please keep in mind that we are working through a large volume of accepted articles, so please give us a few days to review your paper and let you know the next and final steps.

Lastly, if your institution or institutions have a press office, please let them know about your upcoming paper now to help maximize its impact. If they'll be preparing press materials, please inform our press team within the next 48 hours. Your manuscript will remain under strict press embargo until 2 pm Eastern Time on the date of publication. For more information, please contact onepress@plos.org.

You will receive an invoice from PLOS for your publication fee after your manuscript has reached the completed accept phase. If you receive an email requesting payment before acceptance or for any other service, this may be a phishing scheme. Learn how to identify phishing emails and protect your accounts at https://explore.plos.org/phishing.

If we can help with anything else, please email us at customercare@plos.org.

Thank you for submitting your work to PLOS ONE and supporting open access.

Kind regards,

PLOS ONE Editorial Office Staff

on behalf of

Dr. Simone Ranaldi

Academic Editor

PLOS One
